# Impact of COVID-19 on Antibiotic Stewardship, Antimicrobial Resistance, and Prescribing Habits at Two General Hospitals in Abu Dhabi: A Retrospective Analysis

**DOI:** 10.7759/cureus.69170

**Published:** 2024-09-11

**Authors:** Kanika Vats, Kuldeep Singh, Seema Oommen

**Affiliations:** 1 Department of Research and Development, Healthcare Technical and Compliance Directorate, Emirates Classification Society (TASNEEF), Abu Dhabi, ARE; 2 Department of Management, School of Commerce and Management, Om Sterling Global University, Hisar, IND; 3 Department of Laboratory, Burjeel Medical City CoLaB, Abu Dhabi, ARE

**Keywords:** antibiotic resistance, antibiotic stewardship, antibiotic utilization, clinical outcomes, covid-19 pandemic, infection control, intervention strategies, prescribing practices, surveillance and monitoring

## Abstract

Introduction

The COVID-19 pandemic has overwhelmingly affected healthcare systems, particularly in the area of antibiotic management. The surge in antimicrobial use to address secondary bacterial infections in COVID-19 patients has heightened concerns about overuse and antimicrobial resistance (AMR). This study examined the pandemic's effect on the antibiotic stewardship program (ASP) at two general hospitals in Abu Dhabi, focusing on changes in prescribing practices, adherence to stewardship guidelines, resistance trends, and overall health system impact. The present retrospective study evaluated shifts in antibiotic consumption, compliance with stewardship practices, and broader healthcare implications. The aim is to assess the pandemic's impact, identify improvement areas, and provide insights to enhance the ASP in addressing the global AMR crisis.

Methods

This retrospective review assessed electronic medical records from two general hospitals in Abu Dhabi over a 24-month period, from January 2019 to December 2020. It included pre-COVID-19 data from 2019 and data from 2020 during the COVID-19 surge. The study focused on patients aged 25 to 40 years with respiratory tract infections, urinary tract infections, ventilator-associated pneumonia, and nosocomial infections, identified using predefined ICD-10-CM (International Classification of Diseases, Tenth Revision, Clinical Modification) codes. Patients with COVID-19 diagnoses and those undergoing surgical procedures were excluded. Key metrics compared data from 2019 and 2020 to assess changes in clinicians' prescribing practices, antibiotic usage, ASP interventions, and their impact on the healthcare system.

Results

The COVID-19 pandemic influenced antibiotic use and resistance trends, leading to longer hospital stays (3.86 days in 2019 vs. 4.29 days in 2020) and increased use of duplicate anaerobic therapy (4.58% in 2019 vs. 5.71% in 2020). From 2019 to 2020, the average duration of antibiotic therapy decreased from 6.23 days to 5.24 days, but empirical therapy without sufficient evidence rose. The average length of treatment increased (2.87 days in 2019 vs. 3.28 days in 2020), and there was a rise in the use of antibiotics for viral and fungal infections, with cases growing from 17.08% in 2019 to 22.38% in 2020. Despite modest improvements in stewardship practices in 2020, AMR challenges persisted. These results underscore the need for enhanced stewardship programs and continued research to address the ongoing impact on antibiotic prescribing and resistance.

Conclusion

The COVID-19 pandemic increased antibiotic use and altered resistance patterns. Although stewardship practices improved, AMR challenges remained. Enhanced stewardship programs and ongoing research are essential to mitigate these effects and improve antibiotic management.

Recommendation

To address changes in antibiotic use and resistance during the COVID-19 pandemic, it is recommended to strengthen ASPs to adapt to new prescribing trends, ensure adherence to evidence-based practices, provide ongoing education for clinicians, invest in research on long-term resistance impacts, and enhance data tracking and monitoring systems.

## Introduction

Antimicrobial resistance (AMR) was already identified as a critical global emergency long before the COVID-19 pandemic, responsible for approximately 700,000 deaths annually. If current trends continue, AMR is projected to result in 10 million deaths per year by 2050 [1].

AMR is driven by the overuse and misuse of antimicrobial drugs, including antibiotics, antivirals, and antifungals. Globally, over half of all antibiotics are prescribed, distributed, or sold inappropriately. In 80% of countries in the Americas, antibiotics can be purchased without a prescription [2].

The COVID-19 pandemic has exacerbated the global AMR crisis by increasing antibiotic use among COVID-19 patients, disrupting infection prevention and control in overstretched healthcare systems, and diverting resources away from AMR monitoring and response. Consequently, it is crucial to urgently focus on AMR containment efforts and enhance support for detecting, characterizing, and swiftly addressing emerging AMR threats [3].

Although many countries in the region have made substantial progress since 2015 in developing and implementing National Action Plans on AMR through a One Health approach, the COVID-19 emergency shifted priorities. This diversion of human and financial resources from AMR surveillance and response efforts to the COVID-19 response impacted the ongoing AMR initiatives [4].

The COVID-19 pandemic has significantly burdened healthcare systems across the region, heightening the demand for healthcare professionals, intensive care unit beds, and respiratory support such as ventilators. The extended emergency has drained resources and disrupted infection prevention and control practices, resulting in a rise in infections within healthcare settings [5,6].

Despite a rise in antimicrobial prescribing and use, particularly among COVID-19 patients, antimicrobial stewardship programs have not been significantly strengthened as part of the emergency response. Additionally, resources for AMR laboratory surveillance - crucial for accurately assessing the impact of COVID-19 on AMR trends - have also been redirected to support the COVID-19 response [7-9].

The United Arab Emirates (UAE) reported its first case of novel coronavirus on January 29, 2020 [10], and COVID-19 was officially declared a pandemic by global health authorities on March 11, 2020 [11]. In the UAE, the number of cases steadily increased, with the exception of a few months where there was a temporary decline [12].

The health regulator in Abu Dhabi is actively collaborating to combat AMR and enhance the Emirate's resilience. This commitment is evident through initiatives that prioritize research and prevention in emerging infectious diseases and AMR [13]. Accordingly, this study aligns with Abu Dhabi's proactive approach to AMR by examining the impact of COVID-19 on antibiotic stewardship, clinicians' prescribing habits, and the overall effect on the health system among non-surgical and non-COVID hospitalized patients at two general hospitals in the Emirate. The aim is to understand how the pandemic has impacted these practices, contributing to Abu Dhabi's strategic priorities of a healthy population, improved healthcare access and quality, and a resilient and efficient health system [14].

## Materials and methods

This retrospective observational record/data-based study was conducted in two general hospitals from March 2023 to February 2024. Ethical approvals were secured from both the Institutional Review Board of Burjeel Holdings (BH/REC/039/22) and the Department of Health, Abu Dhabi (DOH/CVDC/2023/512).

Study population

The study included all inpatients aged 25 to 40 years who met the eligibility criteria, from January 2019 to December 2020.

Inclusion and exclusion criteria

This study included patients' diseases and medical conditions as documented by clinicians in electronic medical records, identified using ICD-10-CM (International Classification of Diseases, Tenth Revision, Clinical Modification) codes [15]. The focus was on conditions related to the respiratory tract, genitourinary system, nosocomial infections, and other pertinent areas.

Surgical cases, COVID-19-positive cases, patients under 25 or over 40 years of age, and those with other diagnoses were excluded from the study criteria.

Data collection

The data collection process involved several stages. First, electronic medical records (EMRs) were searched using predefined ICD codes, and discharge reports from January 2019 to December 2020 were retrieved for case identification. In the screening stage, duplicate and non-eligible cases were removed using Excel (Microsoft Corporation, Redmond, WA) functions, leaving only records with relevant infections. Next, eligibility was confirmed by sorting records to verify age criteria, with any inconsistencies resolved through further review or consultation with healthcare providers. Data extraction followed, using a standardized Excel template to gather anonymized patient information on demographics, diagnoses, antibiotic use, resistance patterns, and outcomes. Finally, during data cleaning, the dataset was carefully reviewed, errors were corrected, and missing values were addressed to ensure it was ready for analysis.

**Figure 1:**
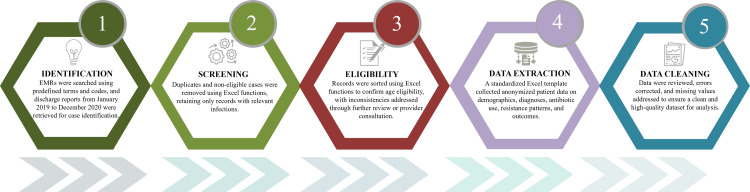
Data collection methodology. EMR: electronic medical records. Image credits: Kanika Vats.

The steps outlined in Figure 1 were used to ensure thorough, consistent, and transparent data collection, enhancing the reliability and validity of the research findings.

This data collection approach was adopted to mitigate several biases: selection bias by including both primary and secondary diagnoses, referral bias by capturing relevant cases regardless of their primary admission reason, observer bias by minimizing subjective judgments, confirmation bias by not prioritizing primary diagnoses, and exclusion bias by ensuring patients with significant secondary conditions were not omitted.

Sampling method

**Figure 2:**
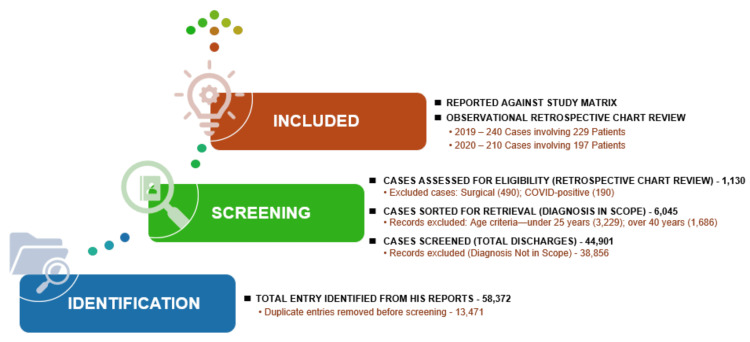
Study case selection process – 2019 and 2020. HIS: Hospital Information System. Image credits: Kanika Vats.

Figure 2 depicts the steps taken to ensure a thorough, unbiased, and accurate selection of cases for the study.

Sample size

A total of 240 cases were analyzed, including 229 patients from 2019 and 210 cases (197 patients) from 2020. The 2019 cohort consisted of 70 males and 170 females, with mean ages of 32.37 years for males and 31.91 years for females. The 2020 cohort included 85 males and 125 females, with mean ages of 32.3 years for males and 31.9 years for females. The study covered 54 nationalities.

Metrics for evaluating study outcomes

The study explores how COVID-19 has affected antibiotic prescribing patterns, the conduct of antibiotic stewardship in an overwhelmed healthcare environment, and the overall impact on the health system. Tables 1-3 outline the key metrics and evaluation methods for each objective, based on local and international guidelines [16-20] for establishing an antibiotic stewardship program (ASP). The objectives are as follows: objective I: effects of COVID-19 on clinicians' prescribing practices and antibiotic use; objective II: impact of COVID-19 on antibiotic stewardship interventions; objective III: impact of COVID-19 on the healthcare system performance.

**Table 1:** Key metrics and assessment methods for objective I. LoT: length of treatment; MAR: medication administration records; DoT: days of therapy. Table Credits: Kanika Vats.

Key metrics	Assessment method
Patient-specific analysis of antibiotic use: inpatient admissions, discharge, and total duration (days)	For each case in 2019 (240 cases) and 2020 (210 cases), the total number of antibiotic days during hospitalization, at discharge, and overall was recorded. The duration of antibiotic use at discharge was noted from the discharge prescription. The results from both years were then compared to identify trends.
Patient-specific antibiotic exposure: LoT (days)	Each case from 2019 (240 cases) and 2020 (210 cases) was evaluated to record the LoT in calendar days for antibiotic therapy, with averages calculated to provide patient-specific data. These values were then compared to assess the impact of COVID-19 on treatment duration.
Selection of antibiotic agents	The MAR for all cases from both years was reviewed to identify occurrences of simultaneous administration of multiple antibiotics for anaerobic therapy. These findings were then compared to evaluate the impact of COVID-19 on the selection of antibiotic agents (n = 240 cases for 2019 and 210 cases for 2020).
Antibiotic use in viral and fungal infections	MAR and laboratory results of all cases were analyzed to identify suspected or confirmed viral and fungal infections, reporting findings regardless of antibiotic use during hospitalization, at discharge, or both, to study practice trends and evaluate the impact of COVID-19 (n = 240 cases for 2019 and 210 for 2020).
Use of unreported antibiotics	All cases were evaluated to assess the use of evidence-based antibiotics by comparing microbiological culture reports (susceptibility/resistance) with MAR and antibiograms. The results were analyzed to determine the impact of COVID-19 on the use of unreported antibiotics (n = 240 cases for 2019 and 210 for 2020).
Antibiotic administration decisions during hospitalization	Antibiotic administration data from MAR were analyzed to identify instances where no antibiotics were prescribed during the patient’s stay, indicating conservative management, and to assess the continuity of this practice during the COVID-19 pandemic (n = 240 cases for 2019 and 210 for 2020).
Antibiotic selection: empirical vs. directed therapy	Eligible cases were evaluated, and comparative results were analyzed to determine whether the antibiotic selection at admission was empirical or directed and to assess the impact of COVID-19 on these decisions. This analysis excluded cases where interventions were made during hospitalization or after microbiological culture reports were released (n = 220 cases for 2019, excluding 20 cases with no antibiotic use during hospitalization, and 186 cases for 2020, excluding 24 cases with no antibiotic use).
Accuracy of empirical antibiotic decisions	The accuracy of empirical antibiotic decisions was assessed by comparing microbiological culture reports with MAR to evaluate the impact of COVID-19 on decision-making. The analysis focused on discrepancies such as antibiotic use without corresponding culture growth, non-compliance with organizational antibiograms for resistant antibiotics, and missing susceptibility data on culture reports. The analysis included n = 189 cases for 2019 (excluding 28 cases with no cultures) and n = 137 cases for 2020 (excluding 42 cases with no cultures).
Antibiotic DoT	All cases from 2019 and 2020 were meticulously reviewed to extract and compare antibiotic administration data from the MAR. The calculation determined the rate of DoT per 1,000 patient days using the following formula: Antibiotic DoT rate = (total quarterly antibiotic DoT/total quarterly patient days) × 1,000. In this context, "Antibiotic DOT" refers to the total number of antibiotic days accumulated by all patients in the facility during each quarter. "Patient days" represents the total number of days patients were hospitalized, including those within the study's scope. The results from both years were compared to evaluate the impact of COVID-19 on antibiotic utilization.

**Table 2:** Key metrics and assessment methods for objective II. Table Credits: Kanika Vats.

Key metrics	Assessment method
Review of antibiotic therapy for inpatients >3 days: escalations, de-escalations, and transitions	Eligible cases were further analyzed to understand practice trends in antibiotic therapy for inpatients with stays longer than three days. This evaluation considered therapy adjustments such as escalations, de-escalations, transitions from intravenous to oral administration, and discontinuation of antibiotics. Results from 2019 and 2020 were compared to assess the impact of COVID-19 on these practices (n = 85 cases for 2019, excluding one case with no antibiotic use, and n = 91 cases for 2020, excluding one case with no antibiotic use).
Review of antibiotic therapy at discharge for stays >3 days: escalations, de-escalations, and transitions	Eligible cases were further analyzed to identify trends in antibiotic therapy at discharge. This assessment focused on modifications such as therapy escalation, de-escalation, transitioning from intravenous to oral administration, and discontinuation of antibiotics, using the 48–72-hour window when microbiological culture results are typically available. Results from 2019 (n = 85 cases, excluding one with no antibiotic use) and 2020 (n = 91 cases, excluding one with no antibiotic use) were compared to evaluate the impact of COVID-19 on these practices.
Antibiotic prescribing decisions at discharge	Data from medication administration records for all cases in both years were reviewed to identify instances where antibiotics were not prescribed at discharge (n = 240 cases for 2019 and n = 205, excluding five cases where discharge data were not accessible for 2020). The results were compared to evaluate changes in prescribing practices between the two years.
Selection of intravenous (IV) antibiotics at discharge	Cases, excluding those without any antibiotic treatment, were closely examined to assess the prescription of IV antibiotics at discharge. Data were collected from discharge prescriptions in the electronic medical records. The findings from 2019 and 2020 were compared to evaluate any changes in the selection of IV antibiotics at discharge during the COVID-19 pandemic (n = 198 cases for 2019, excluding 42 cases with no discharge antibiotics; n = 156 cases for 2020, excluding 49 cases with no discharge antibiotics and five cases with inaccessible data).

**Table 3:** Key metrics and assessment methods for objective III. LoS: length of stay; HAIs: healthcare-associated infections. Table Credits: Kanika Vats.

Key metrics	Assessment method
Average LoS (days)	All cases from both years were documented with their respective admission and discharge dates to calculate the LoS for each patient. The average LoS was then determined and compared across the two years to evaluate the impact of COVID-19 on patient hospitalization duration.
Impact of empiric therapy accuracy on hospital stay duration	Eligible cases from both years were analyzed to assess the impact of empirical therapy accuracy on hospital length of stay. The evaluation combined the total number of completely inaccurate and mixed decisions to determine the overall incidence of inaccurate decisions. This analysis was used to compare the impact of these inaccuracies on hospital stay duration across the two years (n = 189 cases for 2019, excluding 28 cases without cultures, and n = 137 cases for 2020, excluding 42 cases without cultures).
Adverse effects associated with antibiotic therapy	All cases from both years were reviewed to assess the use of probiotics or antidiarrheal medications during hospitalization, identifying patients who may have experienced side effects from extended antibiotic therapy. The findings for 2019 (n = 220, excluding 20 cases without antibiotic administration) were compared to 2020 (n = 186, excluding 24 cases without antibiotic administration) to evaluate any changes in side effect management over the period.
Identification of suspected HAIs or nosocomial infections	Positive cultures with various isolates from both years were examined to identify HAIs, defined as infections occurring at least 48 hours after admission or within 30 days of discharge. Data were collected from microbiological culture reports, including pus swabs, body fluids, sputum (within 72-96 hours), blood (after seven days), and urine (within 48-72 hours), and organized by the date reported (n = 86 cases for 2019 and n = 91 cases for 2020, each with more than three days of inpatient stay). Comparative analysis was performed to assess changes in HAI incidence between the two years.
Tracking 30-day readmissions and their impact	229 patients in 2019 and 197 patients in 2020 were evaluated for 30-day readmissions, concentrating on the financial impact associated with health insurance settlements while excluding staffing and other hospital costs. Comparisons were made to assess changes in readmission patterns and financial implications between the two years.
Mortality rates associated with readmissions	Readmitted cases from both years were assessed to identify any associated mortality rates, using data from discharge summary reports. This comparison highlighted the impact of readmissions on mortality, with n = 4 cases for 2019 and n = 7 cases for 2020.
Antibiotic susceptibility/resistance rates	Institutional antibiograms from both years were analyzed to track trends in antimicrobial resistance and susceptibility to uncover correlations between local antibiotic prescribing practices and infection control measures, highlighting changes in susceptibility and resistance patterns over time.

Statistical analysis

The statistical analysis utilized various Excel functions, including sum, division, average, COUNTIF, multiplication, and percentage. The antibiogram was analyzed using WHONET software, and non-duplicate isolates were selected for the analysis. The percentage of susceptibility was then represented in comparative graphs using Excel's charting functions.

The statistical evaluation of key metrics, resulting in percentage outcomes, was conducted following the methods outlined in Tables 1-3. Microsoft Excel and its functions were the primary tools used for these calculations. The analysis involved using the number of eligible cases that met the criteria for each indicator as the numerator and the total number of cases as the denominator (denoted as "n" against each indicator) in Tables 1-3. This ratio was then multiplied by 100 to generate a percentage figure, representing the findings statistically in the "n (%)" format.

Where applicable, p-values were calculated using Excel's data analysis tool, applying a two-sample t-test assuming unequal variances, with an alpha level set at 0.05.

## Results

Effects of COVID-19 on clinicians’ prescribing practices and antibiotic use

Patient-Specific Analysis of Antibiotic Use

**Figure 3:**
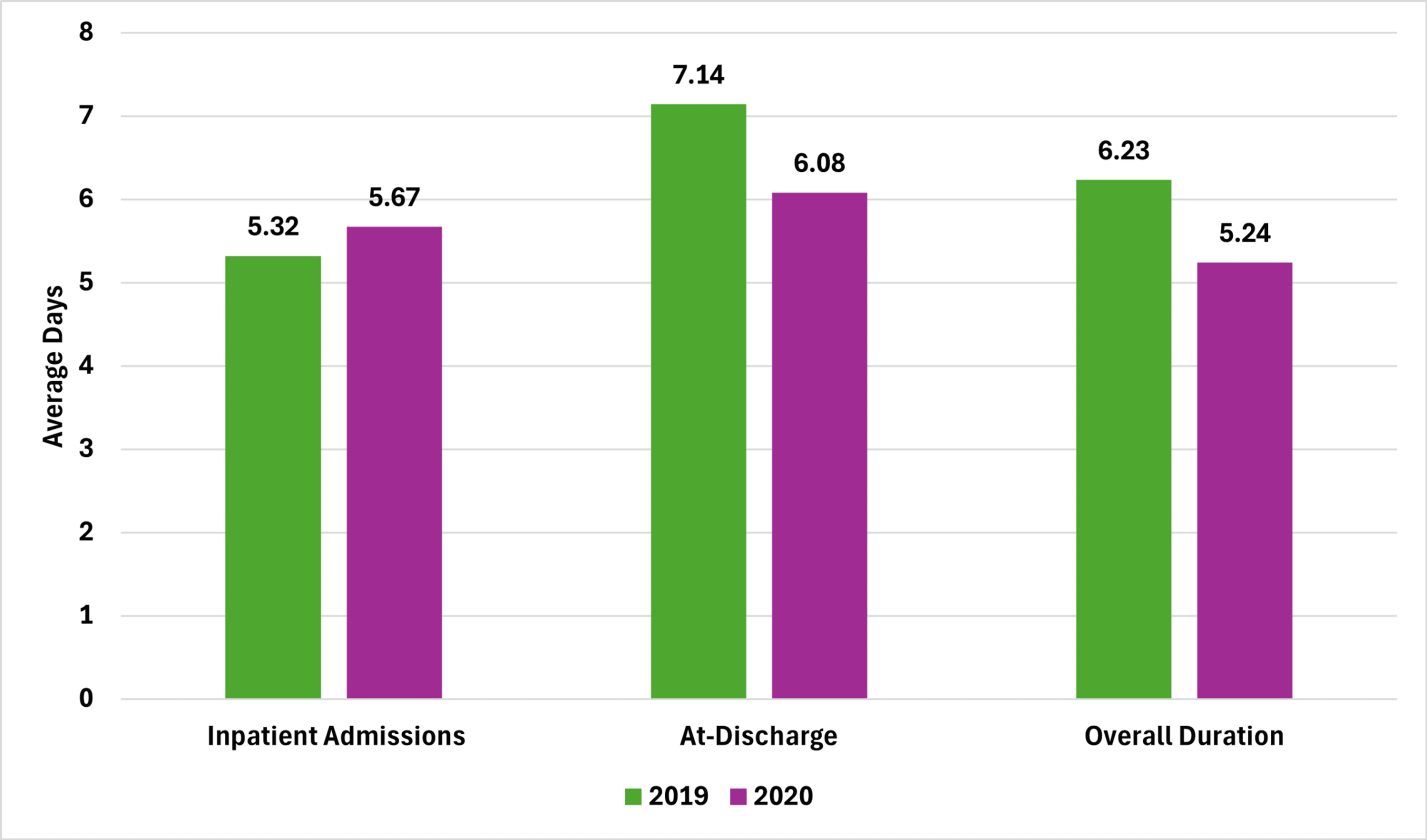
Antibiotic utilization analysis, 2019-2020. Image credits: Kanika Vats.

The patient-wise antibiotic utilization analysis, as illustrated in Figure 3, showed varying trends between 2019 and 2020. For inpatient admissions, the average duration increased from 5.32 days in 2019 to 5.67 days in 2020. However, the average duration of antibiotic use at discharge decreased from 7.14 days in 2019 to 6.08 days in 2020. Overall, the total duration of antibiotic therapy declined from 6.23 days in 2019 to 5.24 days in 2020. The results indicated a cautious increase in inpatient antibiotic use, a reduction at discharge, and an overall decline in therapy duration during COVID-19. This suggests that existing stewardship practices were likely effective even under pandemic conditions, helping to manage antibiotic use and reduce the risk of antimicrobial resistance.

Patient-Specific Antibiotic Exposure (LoT)

The average length of treatment (LoT) increased from 2.87 days in 2019 to 3.28 days in 2020, reflecting a potential shift toward longer treatment durations during this period. This change reflects a more cautious or aggressive approach to treatment, likely driven by uncertainties related to COVID-19 and its associated complications. Additionally, the extended LoT also indicates increased concern over secondary infections, leading clinicians to prescribe longer antibiotic courses.

Selection of Antibiotic Agents

The increase in duplicate anaerobic therapy cases, an ongoing practice from 2019, increased slightly from 11 (4.58%) cases (total = 240) to 12 (5.71%) cases (total = 210) in 2020. This suggests a rise in potentially redundant or overlapping antibiotic use during the COVID-19 pandemic. This trend involves combinations such as meropenem with metronidazole; metronidazole with ceftolozane sulfate and tazobactam; vancomycin, moxifloxacin, and meropenem; and metronidazole with piperacillin and tazobactam, reflecting heightened concerns over severe infections or a more conservative treatment approach.

Antibiotic Use in Viral and Fungal Infections

The increase in antibiotic use for suspected or confirmed viral and fungal infections, an ongoing practice from 2019, rose slightly from 41 (17.08%) cases (total = 240) to 47 (22.38%) cases (total = 210) in 2020. This suggests a rise in potentially inappropriate antibiotic prescribing during the COVID-19 pandemic. The trend indicates that a higher proportion of patients with viral or fungal infections were treated with antibiotics, possibly reflecting increased uncertainty or a more cautious approach by clinicians.

Use of Unreported Antibiotics

Further evaluation of evidence-based antibiotic utilization by comparing microbiological culture reports, medication administration records (MAR), and antibiograms revealed that in 2019, frequently used antibiotics such as macrolides, first-generation cephalosporins, colistimethate, colistin, and metronidazole lacked confirmation in culture results and antibiograms. Azithromycin was also administered but was infrequently reported in culture results. Whereas, in 2020, the introduction of additional antibiotics like fifth-generation cephalosporins, teicoplanin, and doxycycline occurred alongside the continued use of macrolides, colistimethate, and metronidazole, despite the absence of corresponding evidence in culture results or antibiograms. This suggests a potential misalignment between antibiotic prescribing practices and available microbiological evidence, highlighting the need for improved adherence to evidence-based practices to optimize antibiotic use and reduce the risk of resistance.

Antibiotic Administration Decisions During Hospitalization

**Figure 4:**
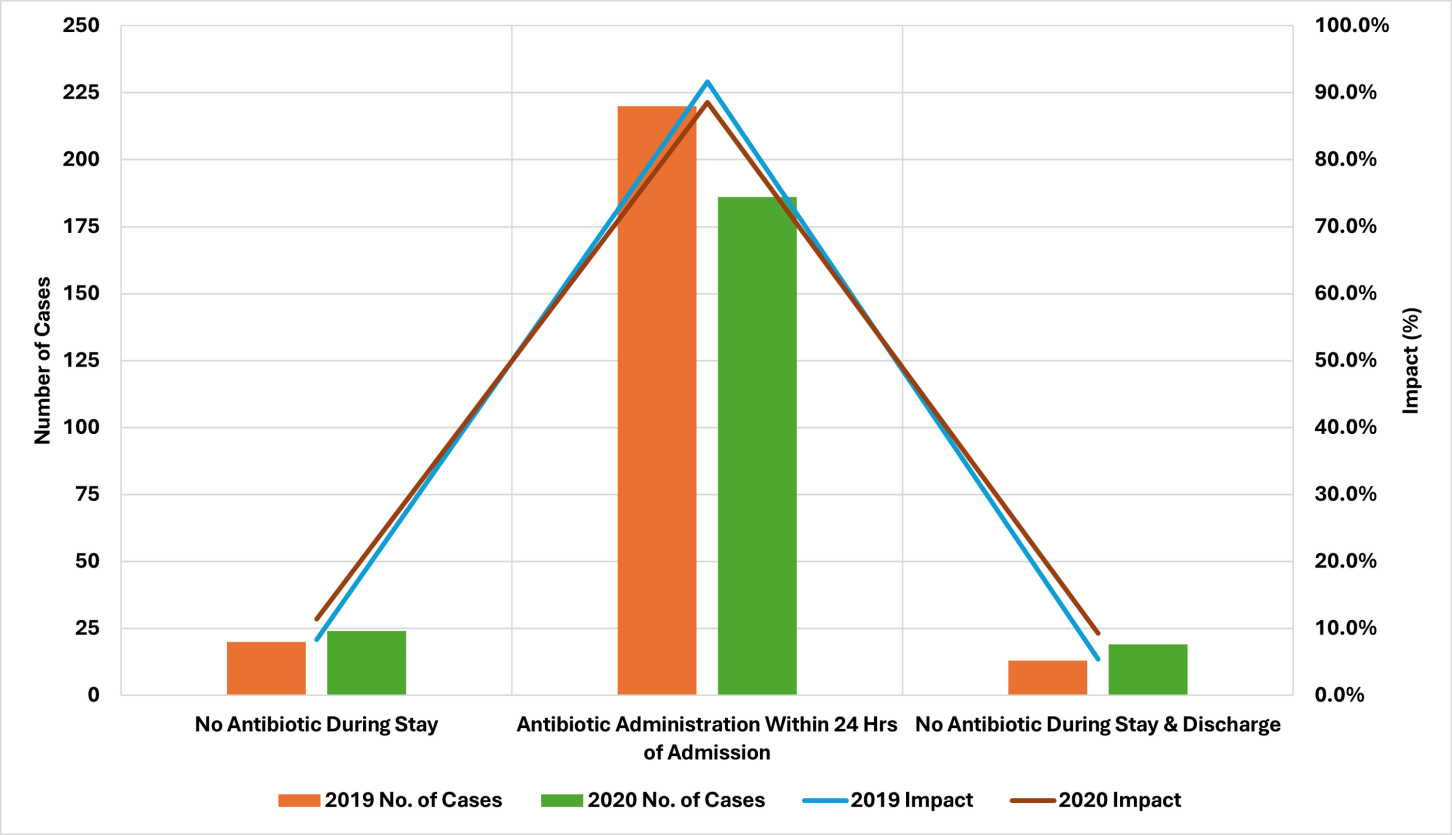
Antibiotic administration practices, 2019 vs. 2020. Image credits: Kanika Vats.

As depicted in Figure 4, the comparison of antibiotic administration practices between 2019 and 2020 reveals notable trends. In 2019, 20 (8.33%) cases (total = 240) did not receive antibiotics during their hospital stay, while this number increased to 24 (11.42%) cases (total = 210) in 2020. The proportion of patients receiving antibiotics within 24 hours of admission decreased from 220 (91.66%) cases (total = 240) in 2019 to 186 (88.57%) cases (total = 210) in 2020. Furthermore, the percentage of patients who did not receive antibiotics during their stay or at discharge rose from 13 (5.41%) cases (total = 240) in 2019 to 19 (9.26%) cases (total = 210) in 2020. These trends suggest a shift toward more selective or cautious antibiotic use, possibly reflecting changes in clinical decision-making during the COVID-19 pandemic. This shift also indicates potentially improved adherence to guidelines for antibiotic use or a greater emphasis on minimizing unnecessary antibiotic exposure, which is important for combating antimicrobial resistance.

Antibiotic Selection: Empirical Vs. Directed Therapy

The comparison of antibiotic selection between empirical and directed therapies highlights a shift from 2019 to 2020. In 2019, empirical antibiotics were used in 217 of 220 cases (98.63%), while directed therapy was chosen in three of 220 cases (1.36%). In 2020, empirical antibiotic use decreased to 179 of 186 cases (96.23%), and the number of directed therapy use increased to seven of 186 cases (3.76%). This trend, excluding interventions based on post-hospitalization microbiological culture reports, demonstrates that while there is a growing adoption of directed therapy, empirical prescribing remains the predominant practice. This continued reliance on empirical therapy highlights the ongoing challenge of balancing immediate treatment needs with the benefits of targeted approaches to optimize patient outcomes and manage antimicrobial resistance.

Accuracy of Empirical Antibiotic Decisions

The review shows a shift in prescribing practices from 2019 to 2020. In 2019, among 189 analyzed cases, 141 (74.60%) cases had inaccurate decisions, with a total of 286 antibiotic selection episodes. Accurate decisions were recorded in 25 (13.2%) of 189 cases, and mixed decisions occurred in 23 (12.16%) of 189 cases, comprising 35 episodes of inaccurate and 25 episodes of accurate prescribing.

In 2020, of 137 cases, 111 (81.02%) cases had inaccurate decisions, with 240 episodes of antibiotic selection. Accurate decisions were observed in 13 (9.48%) of 137 cases, and mixed decisions were noted in 13 (9.48%) of 137 cases, including 25 episodes of incorrect and 16 episodes of correct prescribing. This suggests a decline in the accuracy of empirical decisions over the period, despite the introduction of new therapies and an evolving clinical landscape. The trend highlights the need for improved decision-making processes and adherence to evidence-based practices to enhance the accuracy of empirical antibiotic prescribing and mitigate potential negative impacts on patient outcomes and antimicrobial resistance. Here, an "episode" refers to each instance of antibiotic administration per patient, regardless of details such as duration, dose, or frequency.

Antibiotic DoT

The data indicate fluctuations in days of therapy (DoT) rates for various antibiotics between 2019 and 2020, highlighting correlations with prescribing habits.

The data show fluctuating usage rates of aminoglycosides, azoles, and ansamycins antibiotics across quarters, with significant variability for amikacin and gentamicin, and sporadic increases for co-trimoxazole and rifaximin. Despite these changes, the p-values indicate that none of the antibiotics demonstrated statistically significant (p > 0.05) differences in usage across the quarters.

**Figure 5:**
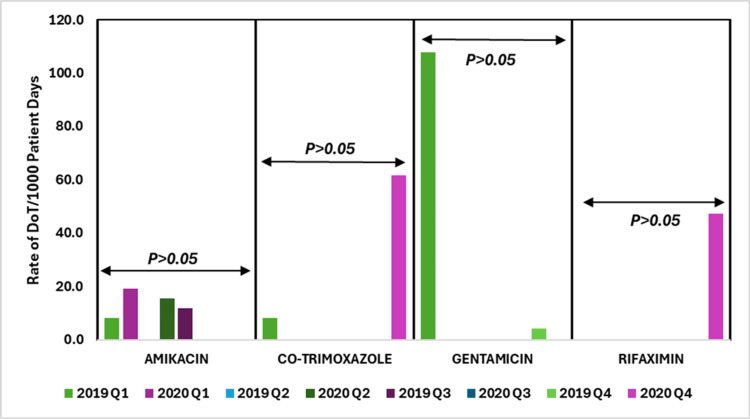
DoT rates/1000 patient days for aminoglycosides, azoles, and ansamycins antibiotics in 2019 vs. 2020. DoT: days of therapy. Image credits: Kanika Vats.

Figure 5 shows the DoT rates per 1000 patient days for aminoglycosides, azoles, and ansamycins antibiotics, comparing data from 2019 to 2020.

The data show variable use of ceftolozane/tazobactam, clindamycin, teicoplanin, and vancomycin hydrochloride across 2019 and 2020. Despite increases in clindamycin and vancomycin during certain quarters of 2020, the p-values (p > 0.05) indicate these changes were not statistically significant. The fluctuations, likely influenced by the COVID-19 pandemic or sustained stewardship practices, were not strong or consistent enough to be statistically validated.

**Figure 6:**
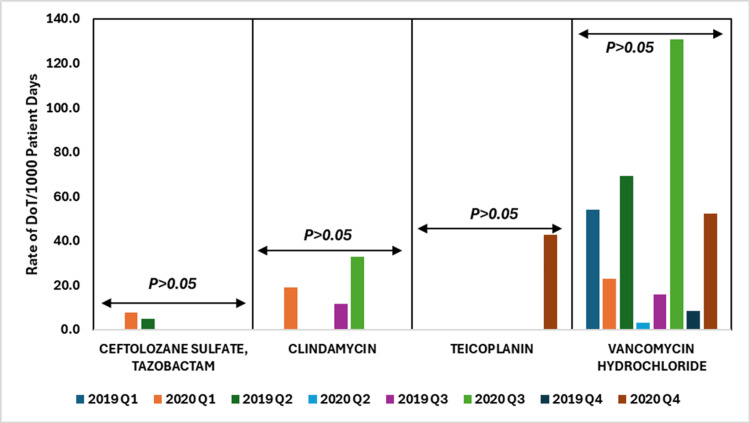
DoT rates/1000 patient days for cephalosporins + β-lactamase inhibitors, lincosamides, and glycopeptides antibiotics in 2019 vs. 2020. DoT: days of therapy. Image credits: Kanika Vats.

Figure 6 illustrates the DoT rates per 1000 patient days for cephalosporins combined with β-lactamase inhibitors, lincosamides, and glycopeptides antibiotics, comparing the data from 2019 to 2020.

The data show varying usage of azithromycin, clarithromycin, erythromycin, and nitrofurantoin across 2019 and 2020. Notably, there were increases in clarithromycin and azithromycin during certain quarters of 2020. However, the p-values (p > 0.05) indicate these changes were not statistically significant, suggesting that the fluctuations, possibly influenced by the COVID-19 pandemic, were not strong or consistent enough to be considered significant.

**Figure 7:**
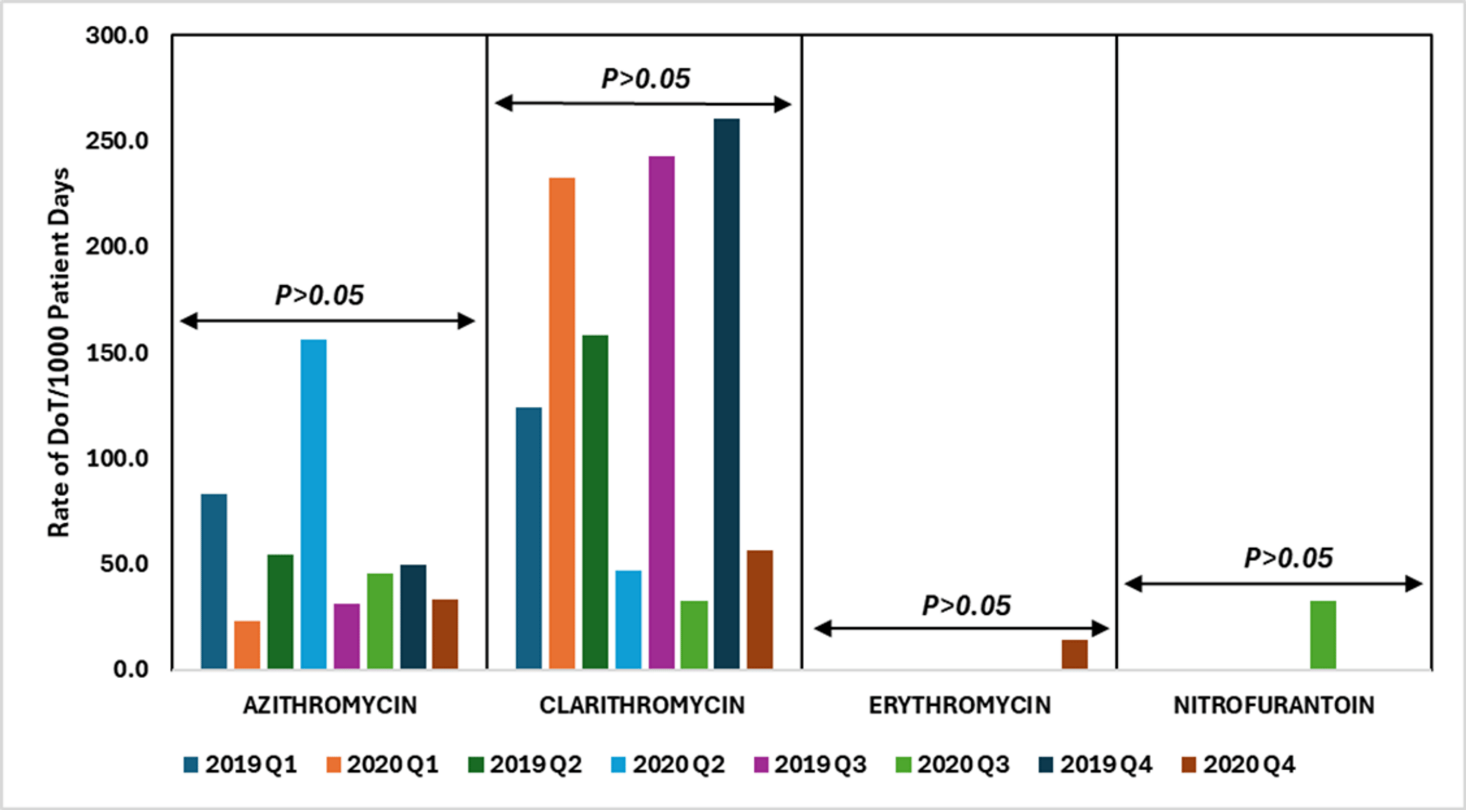
DoT rates/1000 patient days for macrolides and nitrofurans antibiotics in 2019 vs. 2020. DoT: days of therapy. Image credits: Kanika Vats.

Figure 7 depicts the DoT rates per 1000 patient days for macrolides and nitrofurans antibiotics, comparing data from 2019 to 2020.

Co-amoxiclav usage significantly increased in Q1 of 2020 but declined in subsequent quarters, likely due to its role in treating suspected bacterial or secondary infections during COVID-19 and subsequent changes in guidelines and supply chains (p < 0.05). Metronidazole usage increased in Q1, Q3, and Q4 of 2020, likely due to concerns over secondary infections, but the changes were not statistically significant (p > 0.05). Linezolid usage fell to zero in Q2 and Q4 of 2020, with a rise in Q3, reflecting shifting treatment practices during the pandemic, but these changes were not statistically significant (p > 0.05). Fosfomycin usage slightly increased in Q1 of 2020, with no significant changes overall (p > 0.05).

**Figure 8:**
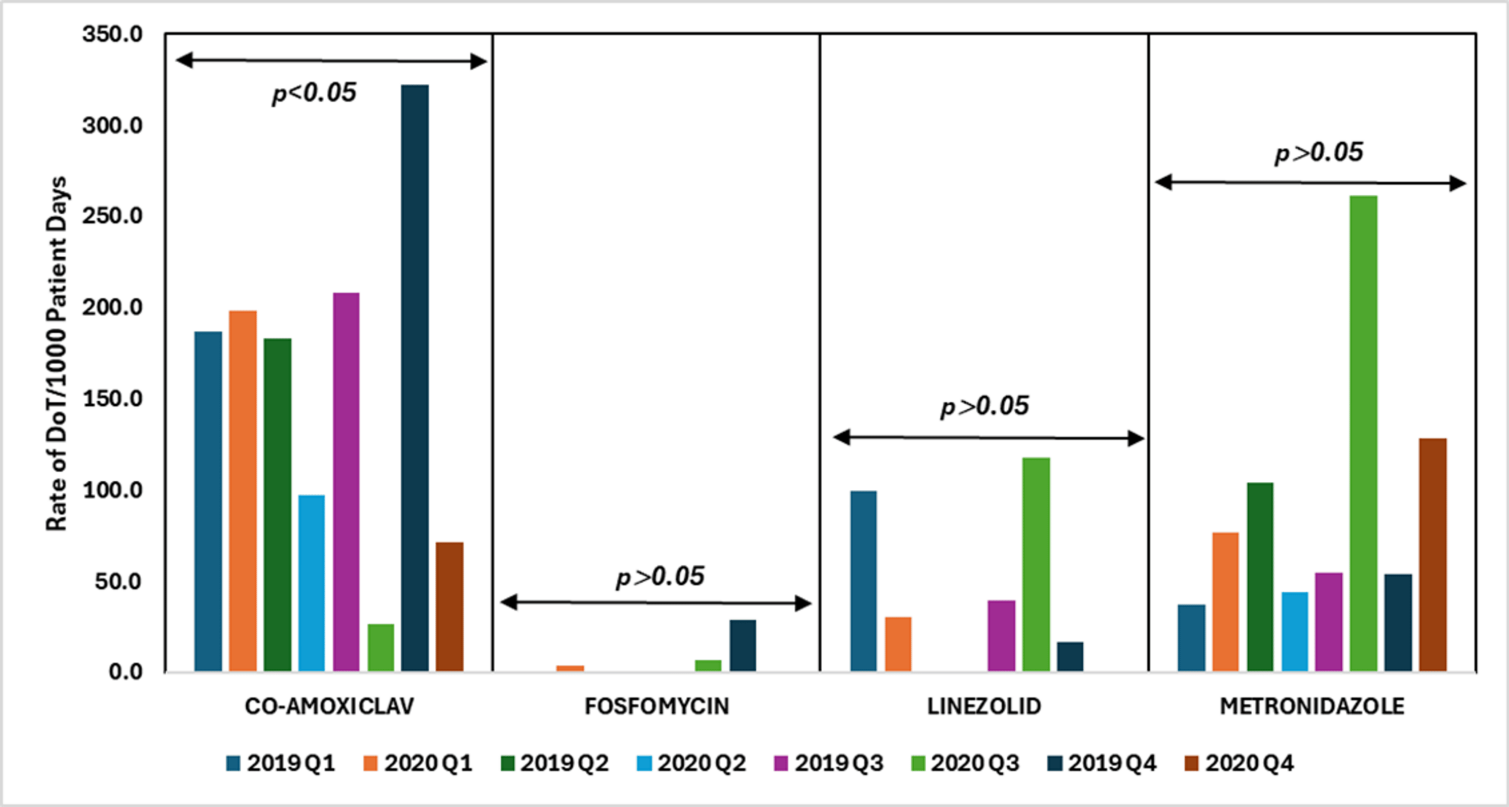
DoT rates/1000 patient days for penicillins + β-lactamase inhibitors, phosphonic, oxazolidinone, and nitroimidazole antibiotics in 2019 vs. 2020. DoT: days of therapy. Image credits: Kanika Vats.

Figure 8 illustrates the DoT rates per 1000 patient days for penicillins combined with β-lactamase inhibitors, phosphonic, oxazolidinones, and nitroimidazole antibiotics, comparing data from 2019 to 2020.

The data revealed fluctuations in the use of ciprofloxacin, colistimethate sodium, colistin, levofloxacin, and moxifloxacin across 2019 and 2020, but none of these changes were statistically significant (p > 0.05). Ciprofloxacin usage increased in Q1, Q2, and Q4 of 2020, while colistimethate sodium and colistin usage were either zero or low with sporadic increases. Levofloxacin showed a substantial rise in Q1 of 2020 but remained variable in other quarters. Moxifloxacin usage was generally low with minor increases. These trends suggest variability in antibiotic use likely influenced by factors such as changes in treatment protocols during the COVID-19 pandemic or potentially sustaining stewardship practices but the fluctuations were not strong enough to be statistically significant.

**Figure 9:**
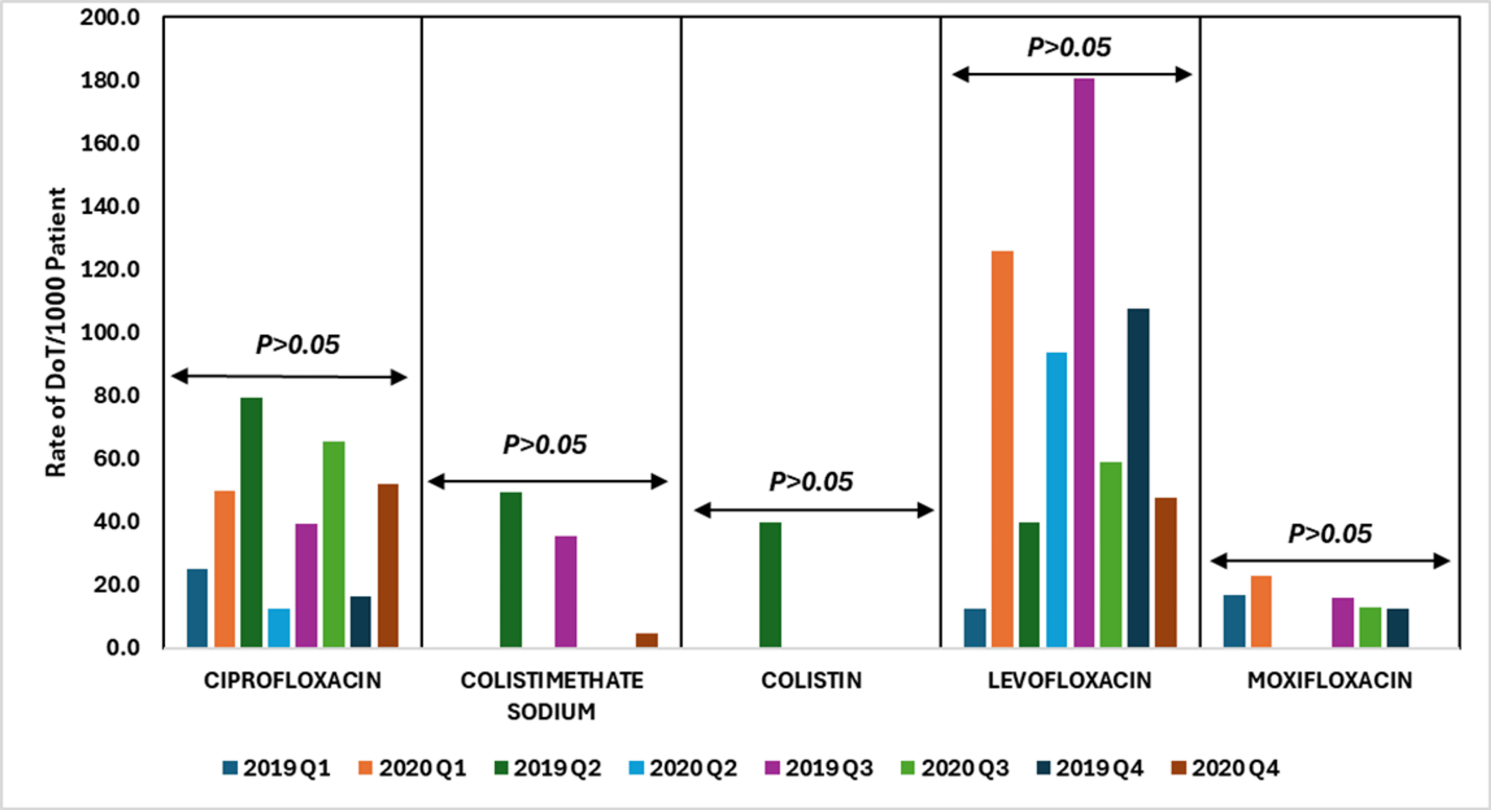
DoT rates/1000 patient days for quinolones and polymyxins antibiotics in 2019 vs. 2020. DoT: days of therapy. Image credits: Kanika Vats.

Figure 9 depicts the DoT rates per 1000 patient days for quinolones and polymyxins antibiotics, comparing data from 2019 to 2020.

Ampicillin showed a notable decline in various quarters, but these changes were not statistically significant (p > 0.05), indicating a trend without strong evidence of a shift. Piperacillin-tazobactam usage, however, increased substantially from 2019 to 2020, with a statistically significant p-value (p < 0.05). This rise suggests a shift toward broader-spectrum antibiotics, potentially driven by emerging clinical needs or evolving resistance patterns.

In contrast, ertapenem's usage dropped to zero across all quarters in 2020, reflecting a significant change in prescribing practices (p < 0.05). This decline may be attributed to improvements in stewardship practices, changes in drug availability, or other factors that are not in the scope of this study. Meanwhile, meropenem usage fluctuated, with an increase observed in Q3 and Q4 of 2020, but these changes were not statistically significant (p > 0.05), indicating stable overall prescribing practices despite the fluctuations.

**Figure 10:**
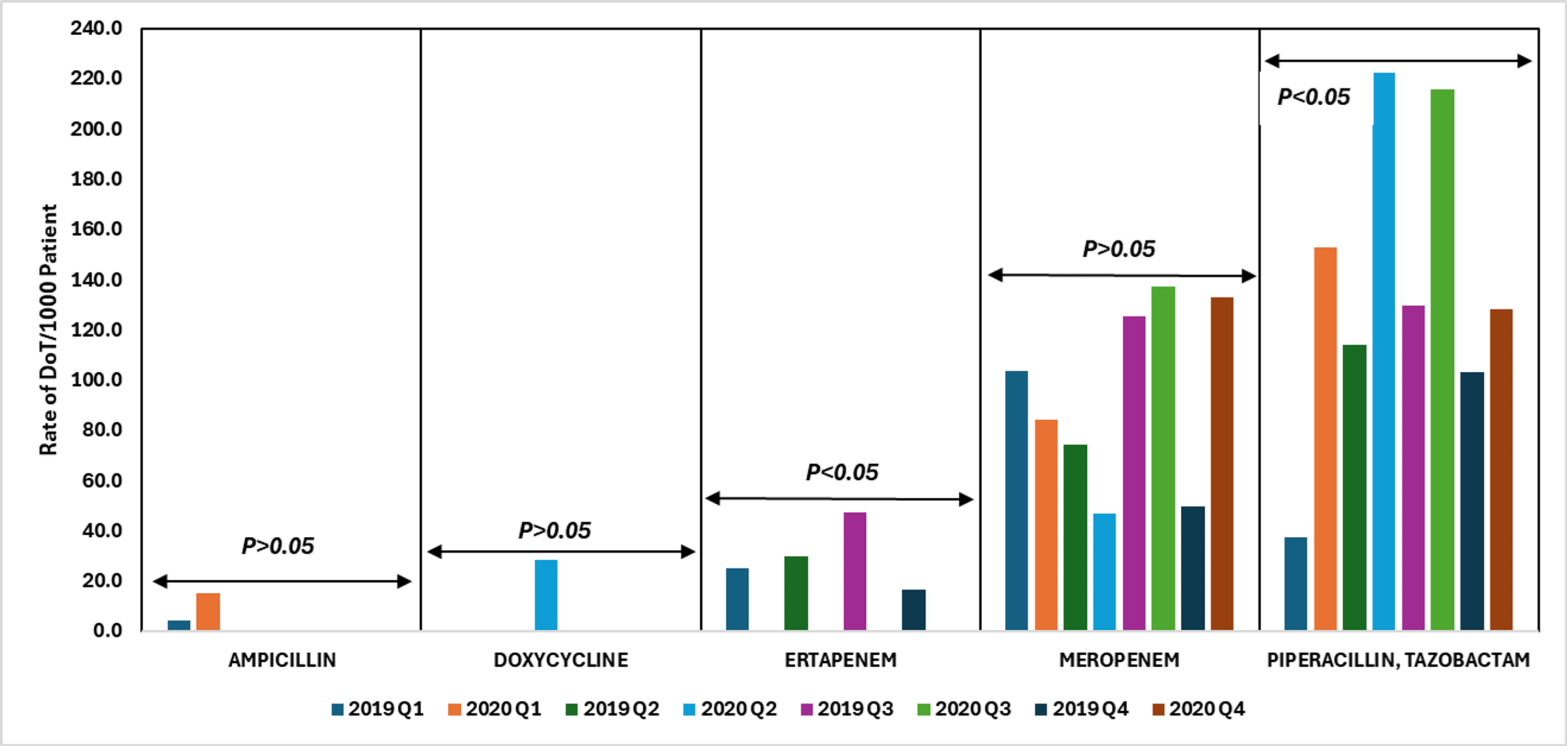
DoT rates/1000 patient days for β-lactam (penicillins), tetracyclines, and β-lactam (carbapenems) antibiotics in 2019 vs. 2020. DoT: days of therapy. Image credits: Kanika Vats.

Figure 10 shows the DoT rates per 1000 patient days for β-lactam (penicillins), tetracyclines, and β-lactam (carbapenems) antibiotics, comparing data from 2019 to 2020.

In 2020, trends in cephalosporin usage showed distinct patterns; however, they were not statistically significant. First-generation cephalosporins, such as cephalexin and cefazolin, experienced a notable decline in usage, with cephalexin seeing zero use across all quarters and cefazolin also significantly reduced. Second-generation cephalosporins showed a slight increase, particularly for cefuroxime in Q3. Third-generation cephalosporins, including ceftriaxone, exhibited stable usage with notable increases observed in Q2 and Q3, while cefotaxime and ceftazidime saw minimal or zero use. Cefixime also saw some usage in Q3, reflecting a potential adaptation in prescribing practices during the pandemic. Fourth-generation cephalosporins, such as cefepime, showed a notable increase in usage, particularly in Q3, with a subsequent decrease in Q4. Overall, the data illustrate a strategic shift toward broader-spectrum cephalosporins, particularly third- and fourth-generation agents, aligning with the increased need for effective treatments during the COVID-19 pandemic.

**Figure 11:**
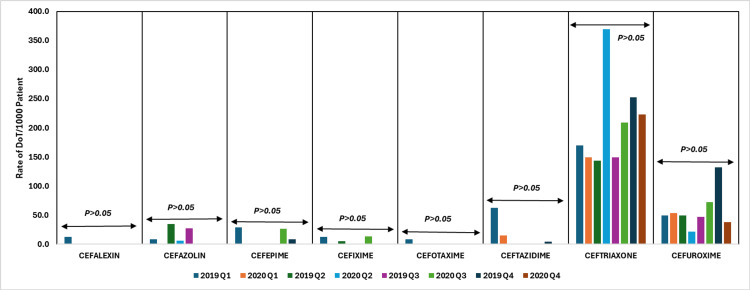
DoT rates/1000 patient days for β-lactam (cephalosporins) antibiotics in 2019 vs. 2020. DoT: days of therapy. Image credits: Kanika Vats.

Figure 11 illustrates the DoT rates per 1000 patient days for β-lactam (cephalosporins) antibiotics, comparing data from 2019 to 2020.

In summary, the COVID-19 pandemic led to prominent shifts in clinician's antibiotic prescribing practices, reflecting both improvements and challenges in stewardship. While overall antibiotic use declined, an increased reliance on broad-spectrum antibiotics and longer treatment durations suggests heightened caution due to pandemic-related uncertainties. The persistence of empirical prescribing and instances of antibiotic use without microbiological confirmation underscore the need for continued focus on evidence-based practices to combat antimicrobial resistance effectively.

Impact of COVID-19 on antibiotic stewardship interventions

Review of Antibiotic Therapy for Inpatients > Three Days

**Figure 12:**
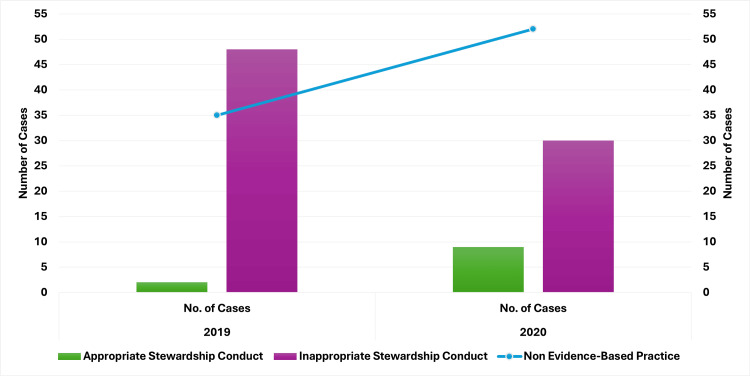
Antibiotic stewardship practices for inpatients (> three days) in 2019 vs. 2020. Image credits: Kanika Vats.

The review revealed a shift in stewardship practices between the two years, as illustrated in Figure 12. In 2019, appropriate stewardship practices were observed in only two (2.35%) of 85 cases. In contrast, inappropriate practices, including the use of multiple sensitive agents, failure to de-escalate or transition from intravenous to oral antibiotics, use of antibiotics not recommended by culture reports, continuation of resistant agents, and lack of therapy adjustment despite negative culture results, were recorded in 48 (56.47%) of 85 cases. Non-evidence-based practices, where culture reports were released on or after discharge or no culture was conducted, were noted in 35 (41.17%) of 85 cases.

In 2020, there was a notable improvement in appropriate stewardship practices, with nine (9.89%) of 91 cases demonstrating adherence to guidelines. The instances of inappropriate stewardship conduct decreased to 30 (32.96%) of 91 cases. However, non-evidence-based practices increased to 52 (57.14%) of 91 cases.

Hence, the results indicated progress in antibiotic stewardship practices between 2019 and 2020. However, persistent inappropriate practices and a rise in non-evidence-based actions reveal ongoing challenges, highlighting the need for further improvements to fully optimize antibiotic use.

*Review of Antibiotic Therapy at Discharge for Stays > Three* *Days*

The stewardship conduct at discharge was reviewed for similar cases, showing notable differences between the two years. In 2019, appropriate stewardship practices were observed in 14 (16.47%) of 85 cases, while inappropriate stewardship practices were noted in 44 (51.76%) of 85 cases. Non-evidence-based practices were identified in 27 (31.76%) of 85 cases. In 2020, there was an improvement in appropriate stewardship practices, increasing to 21 (23.07%) of 91 cases. Inappropriate practices decreased to 29 (31.86%) of 91 cases. However, non-evidence-based practices rose to 41 (45.05%) of 91 cases.

**Figure 13:**
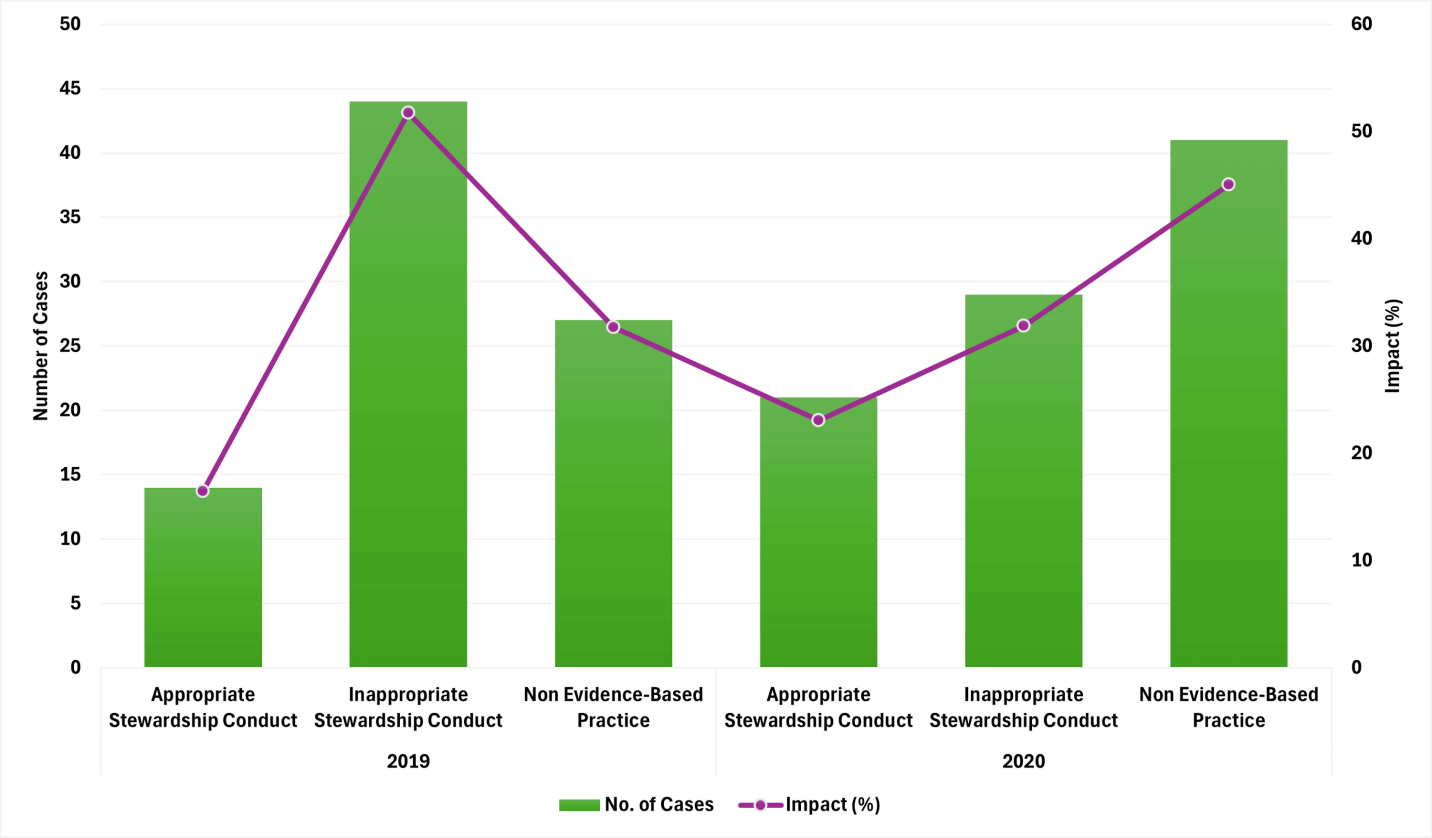
Antibiotic stewardship practices at discharge (> three days) in 2019 vs. 2020. Image credits: Kanika Vats.

The results depicted in Figure 13 indicate that although there have been improvements in following stewardship practices, challenges persist. Specifically, there are ongoing issues with eliminating non-evidence-based practices and maintaining consistent, evidence-driven decision-making at discharge.

Antibiotic Prescribing Decisions at Discharge

The review reflects a trend between 2019 and 2020. In 2019, no antibiotics were prescribed at discharge for 42 cases, representing 17.5% of the total (n = 240). Antibiotics were prescribed in 198 (82.5%) cases (total = 240), with an average of one to three antibiotics per discharge. In seven (2.91%) cases (total = 240), antibiotics were prescribed at discharge even though none were administered during the hospital stay. In 2020, the proportion of patients who did not receive antibiotics at discharge increased to 49 (23.90%) of 205 cases. Antibiotics were prescribed in 156 (76.09%) of 205 cases, excluding five cases where discharge prescriptions were not accessible. Additionally, there were five (2.43%) of 205 cases where no antibiotics were administered during the hospital stay, but they were prescribed at discharge.

These changes suggest a trend toward more judicious antibiotic use at discharge, although a significant number of patients continue to receive antibiotics despite a lack of prior administration, indicating areas for improvement in discharge practices.

Selection of Intravenous Antibiotics at Discharge

The data on the selection of IV antibiotics at discharge revealed an increase from 2019 to 2020. In 2019 (excluding 42 cases with no discharge antibiotics), IV antibiotics were prescribed at discharge in five (2.5%) of 198 cases. This number increased to 15 (9.6%) of 156 cases in 2020, excluding 49 cases where no discharge antibiotic was prescribed and five cases with inaccessible data in the EMR.

This rise may reflect a shift toward more intensive treatment protocols or a response to increased patient complexity. However, it also highlights a need for careful review to ensure that the decision to continue IV antibiotics is evidence-based and necessary, as the increase may also suggest potential areas for overuse or misapplication.

In summary, the study reveals that while there were improvements in antibiotic stewardship practices during the COVID-19 pandemic, substantial challenges remain. The increase in appropriate stewardship actions and the reduction in inappropriate practices from 2019 to 2020 indicate progress. However, the rise in non-evidence-based practices and continued inappropriate antibiotic use at discharge underscore ongoing issues. The increase in IV antibiotic prescriptions at discharge suggests a shift toward more intensive treatment, potentially reflecting patient complexity or overuse. These findings highlight the need for continued efforts to optimize antibiotic stewardship interventions, particularly in ensuring evidence-based practices during and beyond the pandemic.

Impact of COVID-19 on the healthcare system performance

Average LoS (Days)

The average length of stay (LoS) rose from 3.86 days in 2019 (n = 240 cases) to 4.29 days in 2020 (n = 210 cases), highlighting a noticeable increase in hospital stay duration between 2019 and 2020. This rise may indicate increased patient complexity or changes in clinical management practices, potentially influenced by the COVID-19 pandemic. It suggests that more severe cases, extended treatments, and evolving care protocols are contributing to longer hospital stays.

Impact of Empiric Therapy Accuracy on Hospital Stay Duration

The analysis of the impact of empirical therapy decisions on hospital stay length also shows variations between 2019 (n = 189 cases) and 2020 (n = 137 cases). In 2019, the average LoS for patients with accurate empirical decisions was 3.36 days, whereas 4.45 days for those with inaccurate decisions. In 2020, the average LoS increased for both groups, reaching 4.6 days for accurate decisions and 5.13 days for inaccurate decisions.

The rise in LoS for both accurate and inaccurate empirical therapy decisions from 2019 to 2020 indicates a trend toward longer hospitalizations. This suggests that although overall hospital stays increased, the influence of decision accuracy on LoS remained significant, emphasizing the need for enhanced empirical decision-making to better manage hospital stays.

Adverse Effects Associated With Antibiotic Therapy

In 2019, probiotics or antidiarrheal drugs were used in three cases, accounting for 1.36% of the total cases considered (n = 220), excluding the 20 patients who were not administered antibiotics during their hospital stay. In 2020, the number of cases where these treatments were used increased to 19, representing 10.21% of the total cases considered (n = 186), excluding the 24 patients who did not receive antibiotics during their hospitalization. This rise, along with longer hospital stays and treatments, reflects the impact of inaccurate empirical decisions, which contribute to extended care and increased gastrointestinal issues.

Identification of Suspected HAIs or Nosocomial Infections

In 2019, seven (8.13%) of 86 cases were suspected of healthcare-associated infections (HAIs) or nosocomial infections, involving pathogens such as *Escherichia coli*, *Pseudomonas spp.*, *Bacillus spp.*, *Klebsiella pneumoniae*, *Staphylococcus* *coagulase-negative spp.*, and *Enterococcus spp*. In contrast, in 2020, the number increased to 11 (12.08%) suspected cases of 91 cases, with pathogens including *Serratia spp.*, *Acinetobacter spp.*, *Escherichia coli*, *Klebsiella pneumoniae*, and *Candida albicans*. This rise suggests greater infection complexity, potentially influenced by the COVID-19 pandemic's impact on hospital infection dynamics.

Tracking 30-Day Readmissions and Their Impact

In 2019, four patients were readmitted within 30 days of initial care, while in 2020, this number increased to seven. Financially, these readmissions amounted to approximately $18,927 in 2019, which rose to approximately $45,463 in 2020. These figures are based on claims submitted to health payers, excluding staffing and other direct or indirect costs.

This trend suggests potential issues with stewardship practices and emphasizes the need for improved discharge planning and follow-up to reduce readmission rates and their financial impact.

Mortality Rates Associated With Readmissions

The increase in mortality rate among readmitted patients, from four (0%) in 2019 to seven (14.3%) in 2020, highlights the potential link between inadequate stewardship practices and higher mortality risk. This highlights the need for enhanced stewardship and follow-up strategies to improve patient outcomes and reduce readmission-related mortality.

Antibiotic Susceptibility/Resistance Rates

Overall, the data highlight varying trends in antibiotic susceptibility among the studied Gram-positive organisms, with some showing resilience or improvement and others experiencing declining susceptibility.

*Staphylococcus* (coagulase-negative) susceptibility to amoxicillin/clavulanic acid (AMC) decreased from 82% in 2019 to 73% in 2020 and similar trends were observed for cefazolin (CZO), ceftriaxone (CRO), cefuroxime (CXM), and oxacillin (OXA). There was a reduction in susceptibility of ciprofloxacin (CIP), clindamycin (CLI), erythromycin (ERY), levofloxacin (LVX), rifampin (RIF), tetracycline (TCY), and trimethoprim/sulfamethoxazole (SXT) while linezolid (LNZ), gentamicin (GEN), nitrofurantoin (NIT), and vancomycin (VAN) maintained high susceptibility rates.

For *Streptococcus* (beta-hemolytic group B), susceptibility to AMC, ampicillin (AMP), CZO, cefotaxime (CTX), CRO, CXM, LNZ, NIT, meropenem (MEM), piperacillin/tazobactam (TZP), and VAN remained consistently high at 100% for both years. However, susceptibility to CLI showed a slight decrease from 87% in 2019 to 85% in 2020, and TCY remained almost resistant, with only 4% susceptibility for both years.

**Figure 14:**
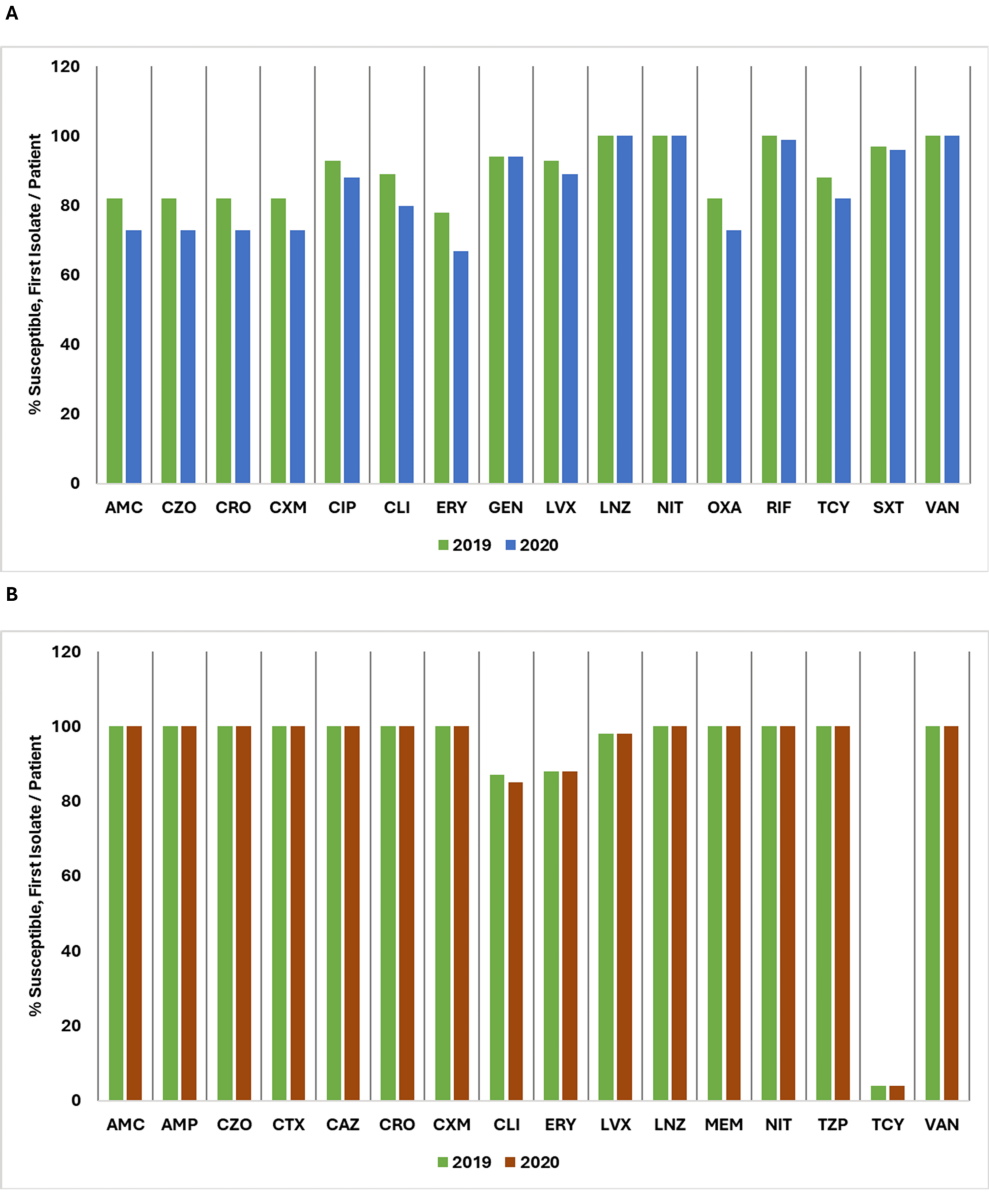
(A) Susceptibility (%) of Staphylococcus (coagulase-negative): 2019 vs. 2020. (B) Susceptibility (%) of Streptococcus (beta-hemolytic group B): 2019 vs. 2020. AMC: amoxicillin/clavulanic acid; AMP: ampicillin; CAZ: ceftazidime; CIP: ciprofloxacin; CLI: clindamycin; CRO: ceftriaxone; CTX: cefotaxime; CXM: cefuroxime; CZO: cefazolin; ERY: erythromycin; GEH: gentamicin-high; GEN: gentamicin; LNZ: linezolid; MEM: meropenem; NIT: nitrofurantoin; OXA: oxacillin; LVX: levofloxacin; RIF: rifampin; SXT: trimethoprim/sulfamethoxazole; TCY: tetracycline; TZP: piperacillin/tazobactam; VAN: vancomycin. Source: Data from institutional antibiogram. Image credits: Kanika Vats.

Figure 14 shows a comparison of the percentage of susceptible isolates per patient for *Staphylococcus *(coagulase-negative) and *Streptococcus* (beta-hemolytic group B) between 2019 and 2020.

For *Staphylococcus aureus*, susceptibility to AMC, CZO, CTX, CRO, and CXM slightly decreased from 76% in 2019 to 74% in 2020. A similar trend was observed for ERY, which declined from 78% to 72%, CLI from 86% to 85%, GEN from 92% to 91%, and OXA from 76% to 73%. Conversely, TCY showed an increase in susceptibility, rising from 85% to 89%.

For *Enterococcus* species*,* susceptibility to AMC slightly decreased from 100% in 2019 to 99% in 2020. A notable decline was observed in TCY from 14% to 7%.

**Figure 15:**
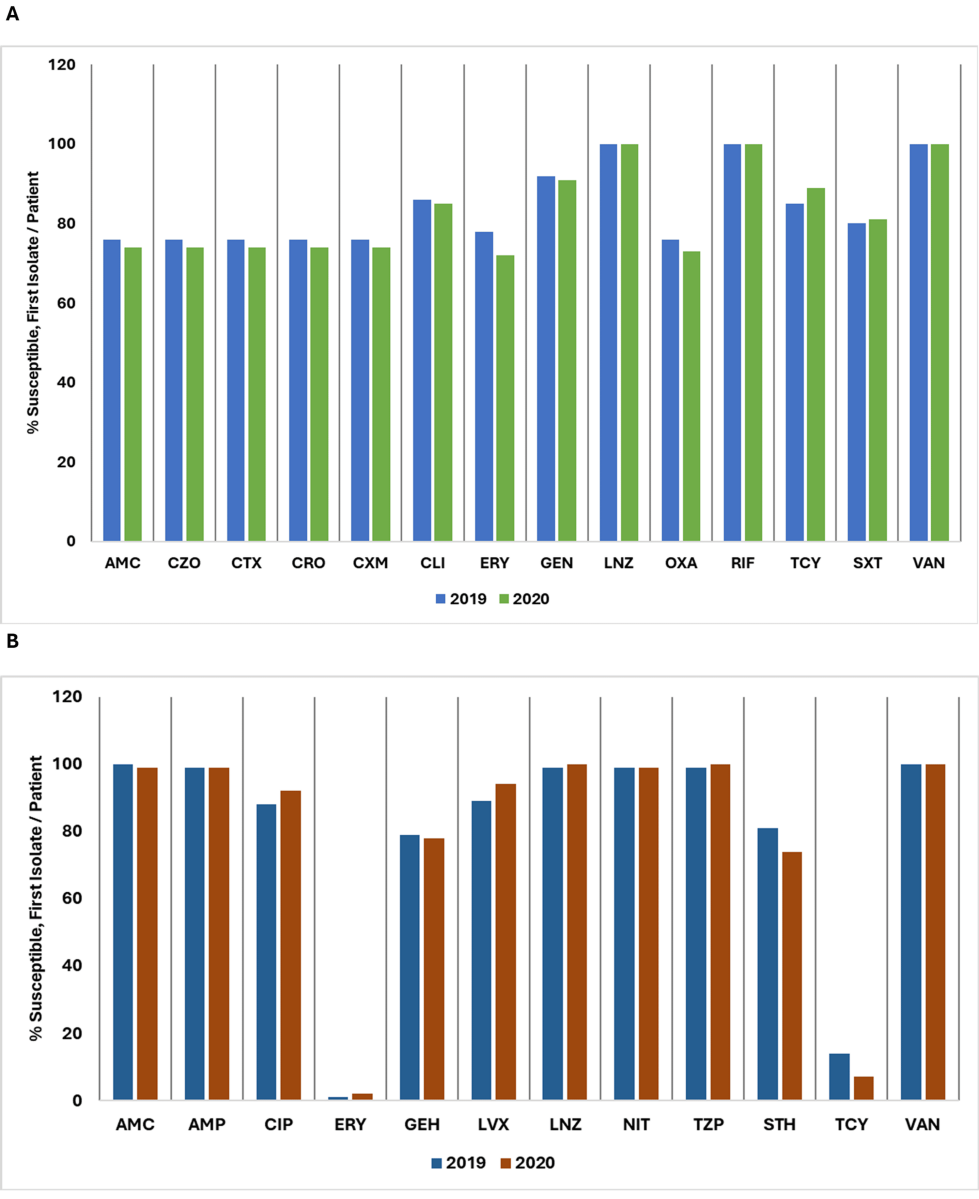
(A) Susceptibility (%) of Staphylococcus aureus: 2019 vs. 2020. (B) Susceptibility (%) of Enterococcus species: 2019 vs. 2020. AMC: amoxicillin/clavulanic acid; AMP: ampicillin; CIP: ciprofloxacin; CLI: clindamycin; CRO: ceftriaxone; CTX: cefotaxime; CXM: cefuroxime; CZO: cefazolin; ERY: erythromycin; GEH: gentamicin-high; GEN: gentamicin; LNZ: linezolid; LVX: levofloxacin; NIT: nitrofurantoin; OXA: oxacillin; RIF: rifampin; STH: streptomycin-high; SXT: trimethoprim/sulfamethoxazole; TCY: tetracycline; TZP: piperacillin/tazobactam; VAN: vancomycin. Source: Data from institutional antibiogram. Image credits: Kanika Vats.

Figure 15 presents a comparison of the percentage of susceptible isolates per patient for *Staphylococcus aureus* and *Enterococcus species*, across the years 2019 and 2020.

For *Streptococcus pneumoniae* and *Streptococcus pyogenes*, there is a lack of data for 2020 to assess susceptibility trends. However, in 2019, susceptibility was below 80% for CLI, ERY, TCY, and SXT for *Streptococcus pneumoniae*, and for ERY and TCY for *Streptococcus pyogenes*.

In 2019 and 2020, the antibiotic susceptibility for Gram-negative organisms also showed varying trends. These data highlight the shifts in antibiotic resistance patterns among Gram-negative bacteria between the two years, indicating both areas of consistent effectiveness and emerging resistance.

For *Escherichia coli*, susceptibility to amikacin (AMK) stayed high, with a slight increase from 97% in 2019 to 98% in 2020. Susceptibilities to AMC, AMP, cefepime (FEP), cefixime (CFM), CRO, CIP, CXM, ertapenem (ETP), fosfomycin (FOS), GEN, imipenem (IPM), LVX, NIT, TZP, MEM, and SXT remained stable or exhibited minor declines. However, susceptibility to CZO dropped significantly from 29% to 12%, and susceptibility to norfloxacin (NOR) decreased from 80% to 56%.

For *Klebsiella pneumoniae*, susceptibility trends showed that AMK increased from 98% to 99%, while AMC remained stable at 84%. FEP showed a slight rise from 82% to 83%, but CFM decreased from 80% to 79%. CXM dropped from 70% to 62%, and NIT decreased from 54% to 38%. Other agents like CTX, ceftazidime (CAZ), CRO, ETP, GEN, IPM, LVX, and MEM remained stable at their respective levels. Notably, susceptibility to NOR decreased significantly from 93% to 63%, and SXT saw a minor increase from 83% to 84%.

**Figure 16:**
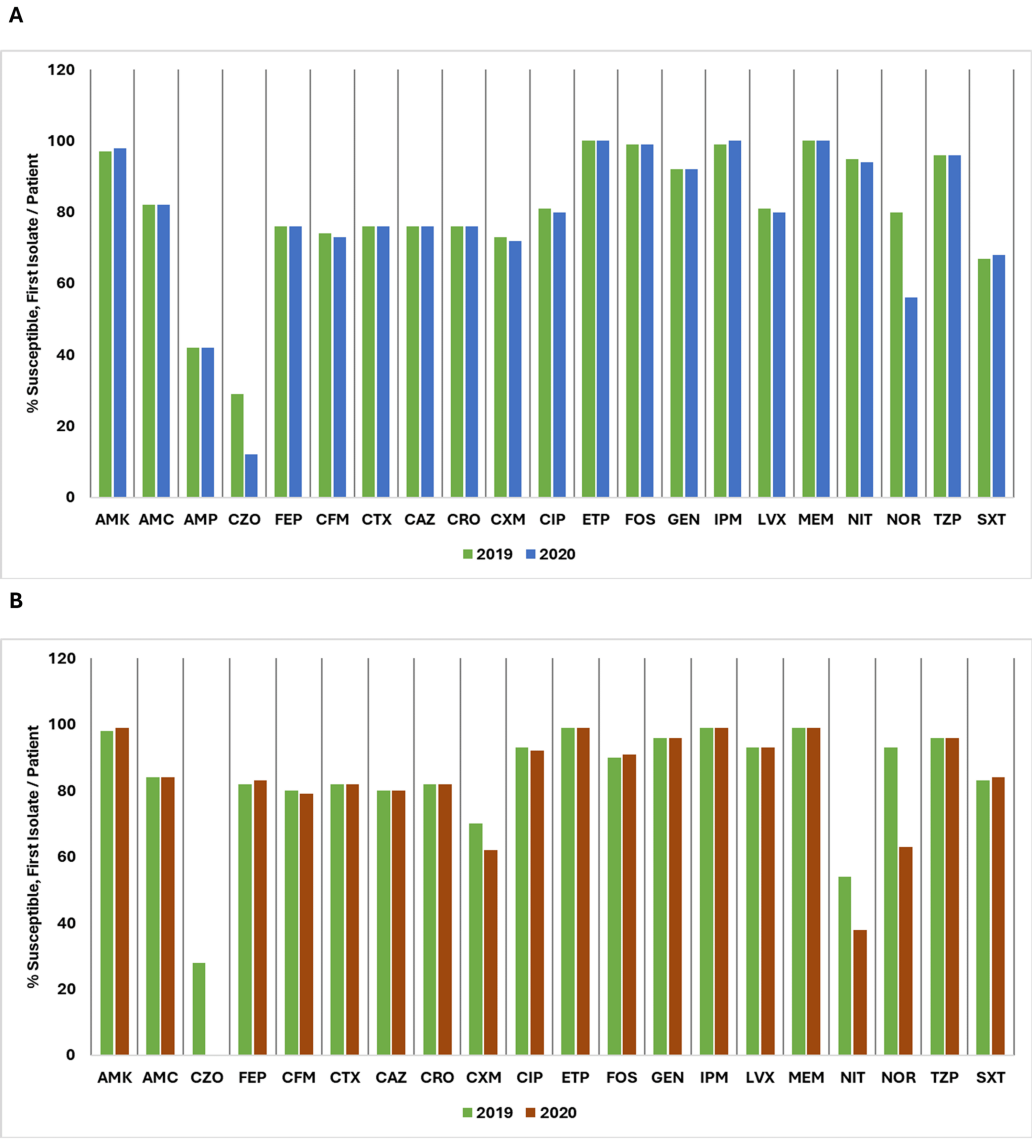
(A) Susceptibility (%) of Escherichia coli: 2019 vs. 2020. (B) Susceptibility (%) of Klebsiella pneumoniae: 2019 vs. 2020. AMK: amikacin; AMC: amoxicillin/clavulanic acid; AMP: ampicillin; CAZ: ceftazidime; CFM: cefixime; CIP: ciprofloxacin; CRO: ceftriaxone; CTX: cefotaxime; CXM: cefuroxime; CZO: cefazolin; ETP: ertapenem; FEP: cefepime; FOS: fosfomycin; GEN: gentamicin; IPM: imipenem; LVX: levofloxacin; MEM: meropenem; NIT: nitrofurantoin; NOR: norfloxacin; SXT: trimethoprim/sulfamethoxazole; TZP: piperacillin/tazobactam. Source: Data from institutional antibiogram. Image credits: Kanika Vats.

Figure 16 depicts a comparison of the percentage of susceptible isolates per patient for *Escherichia coli* and *Klebsiella pneumoniae* between 2019 and 2020.

For *Enterobacter species*, susceptibility data indicate that AMK decreased from 99% to 97%, while FEP improved from 79% to 82%. Susceptibility to NIT decreased from 44% to 34%. Other agents, including CIP, ETP, GEN, IPM, LVX, and MEM, remained stable or showed minor changes. FOS dropped slightly from 86% to 84%, and NOR decreased from 91% to 65%. TZP increased from 92% to 93%, and SXT decreased from 87% to 85%.

For *Proteus species*, susceptibility to AMK was stable at 98% in both 2019 and 2020. The susceptibility to AMC increased from 87% to 89%, and AMP rose from 63% to 66%. FEP, CFM, CTX, CAZ, and CRO showed consistently high susceptibility rates of 93%, 92%, 92%, 93%, and 92%, respectively. CXM saw a slight drop from 87% to 86%, while CIP improved from 86% to 88%. ETP decreased slightly from 100% to 99%. FOS increased from 90% to 92%, and GEN remained stable at 87%. IPM rose from 36% to 44%, and LVX saw a notable increase from 86% to 91%. MEM had a minor decrease from 99% to 98%. NOR dropped significantly from 85% to 68%, and TZP decreased slightly from 100% to 99%. SXT held steady at 75%.

**Figure 17:**
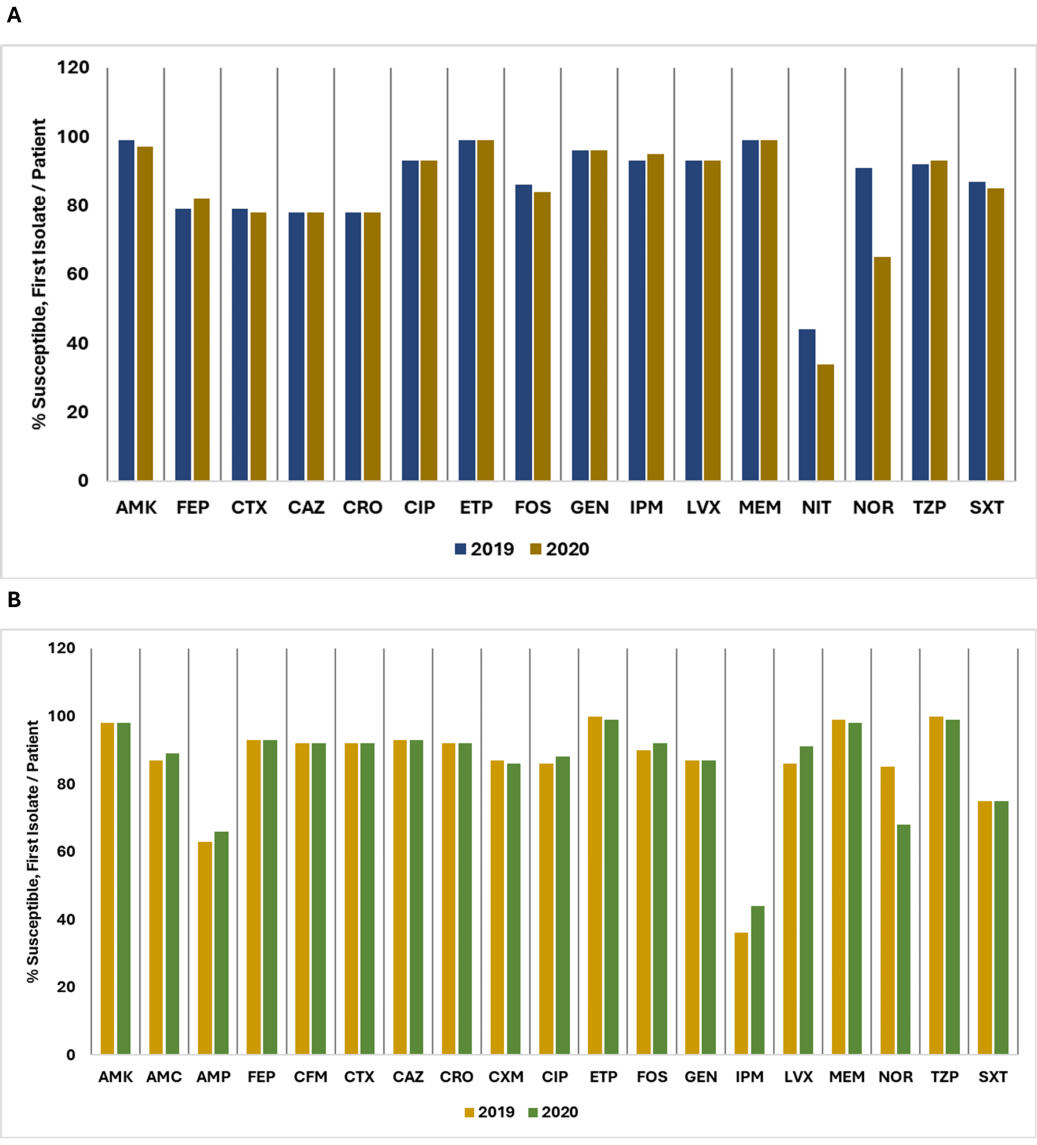
(A) Susceptibility (%) of Enterobacter species: 2019 vs. 2020. (B) Susceptibility (%) of Proteus species: 2019 vs. 2020. AMK: amikacin; AMC: amoxicillin/clavulanic acid; AMP: ampicillin; CAZ: ceftazidime; CFM: cefixime; CIP: ciprofloxacin; CRO: ceftriaxone; CTX: cefotaxime; CXM: cefuroxime; ETP: ertapenem; FEP: cefepime; FOS: fosfomycin; GEN: gentamicin; IPM: imipenem; LVX: levofloxacin; MEM: meropenem; NIT: nitrofurantoin; NOR: norfloxacin; TZP: piperacillin/tazobactam; SXT: trimethoprim/sulfamethoxazole. Source: Data from institutional antibiogram. Image credits: Kanika Vats.

Figure 17 shows a comparison of the percentage of susceptible isolates per patient for *Enterobacter species* and *Proteus species* between 2019 and 2020.

For *Citrobacter species*, susceptibility to AMK rose from 98% in 2019 to 99% in 2020. AMC increased from 76% to 87%, while AMP remained low, rising from 0% to 1%. CZO data for 2020 were unavailable but was 10% in 2019. Susceptibility to FEP improved from 88% to 94%, and CFM increased from 76% to 87%. Both CTX and CAZ rose to 95% and 94%, respectively. CRO improved from 89% to 95%, while CXM slightly decreased from 65% to 63%. CIP and ETP increased to 97% and 100%, respectively. FOS improved from 97% to 99%, with GEN and IPM also rising to 99%. LVX increased from 95% to 97%, and MEM remained stable at 99%. NIT decreased slightly from 89% to 87%, and NOR dropped significantly from 95% to 75%. TZP stayed high at 98%, and SXT improved from 94% to 95%.

For *Serratia species*, susceptibility to AMK increased from 93% in 2019 to 99% in 2020. FEP, CTX, CAZ, and CRO also saw improvements, rising from 90% to 97%, 87% to 97%, 87% to 97%, and 87% to 95%, respectively. CIP increased from 92% to 99%, while ETP rose from 93% to 100%. GEN and LVX improved from 94% to 98% and 92% to 99%, respectively. MEM remained stable at 99%. NOR decreased significantly from 100% to 89%, and SXT improved from 96% to 99%.

**Figure 18:**
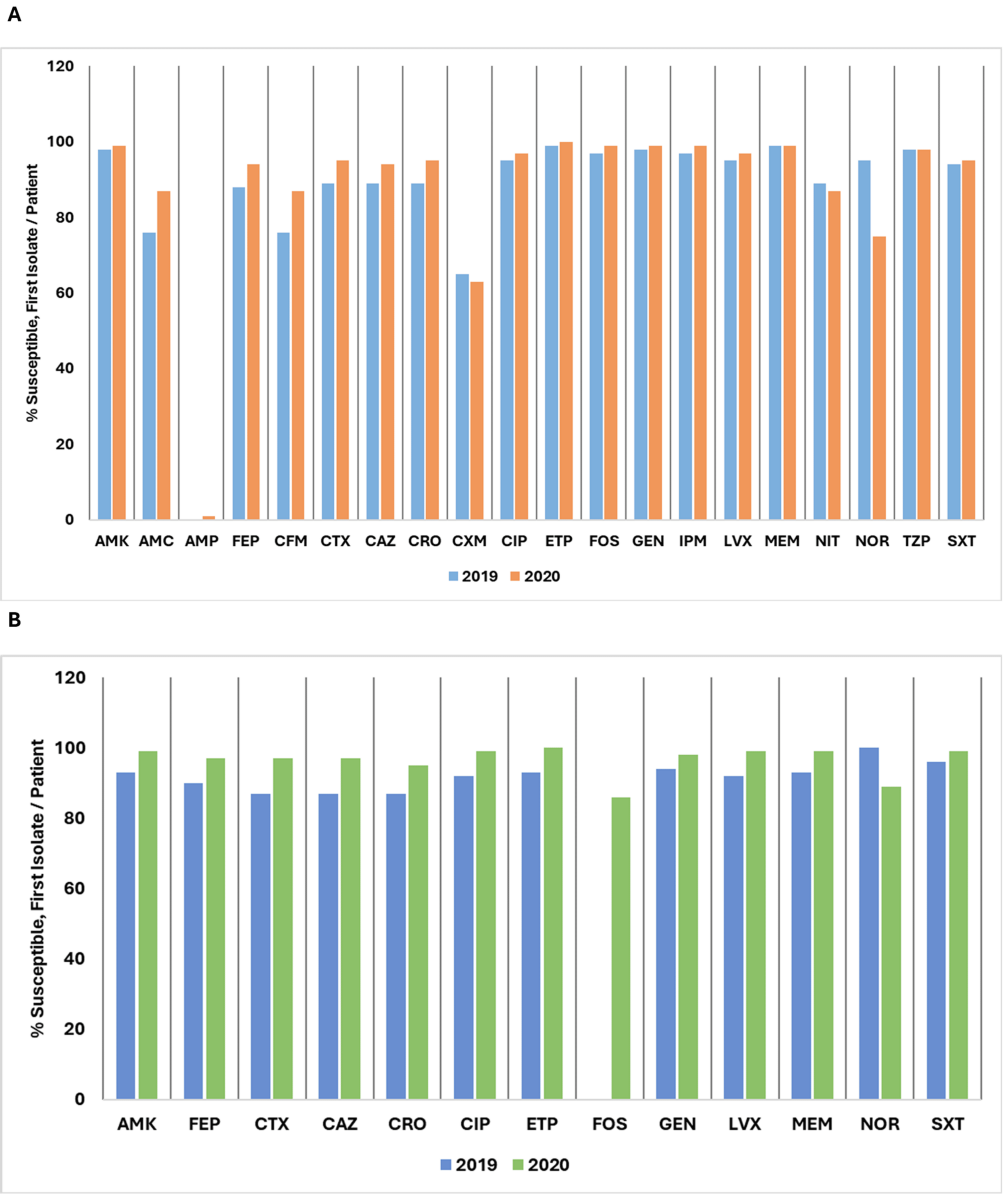
(A) Susceptibility (%) of Citrobacter species: 2019 vs. 2020. (B) Susceptibility (%) of Serratia species: 2019 vs. 2020. AMK: amikacin; AMC: amoxicillin/clavulanic acid; AMP: ampicillin; CIP: ciprofloxacin; CRO: ceftriaxone; CFM: cefixime; CTX: cefotaxime; CAZ: ceftazidime; MEM: meropenem; CXM: cefuroxime; ETP: ertapenem; FEP: cefepime; FOS: fosfomycin; GEN: gentamicin; IPM: imipenem; LVX: levofloxacin; NIT: nitrofurantoin; NOR: norfloxacin; TZP: piperacillin/tazobactam; SXT: trimethoprim/sulfamethoxazole. Source: Data from institutional antibiogram. Image credits: Kanika Vats.

Figure 18 shows a comparison of the percentage of susceptible isolates per patient for *Citrobacter species* and *Serratia species* between 2019 and 2020.

For *Salmonella species*, susceptibility to AMP improved from 69% to 76%. FEP, CTX, and CRO all increased, with FEP at 91%, and CTX and CRO at 94%. CIP rose from 52% to 73%. ETP, IPM, and MEM reached 100% susceptibility, while LVX increased to 73% and NOR reached 74%. SXT improved from 85% to 91%.

For *Pseudomonas species*, susceptibility data show that AMK increased slightly from 97% to 98%. FEP improved from 94% to 95%, while CAZ decreased from 95% to 93%. CIP showed a reduction from 89% to 86%, and LVX decreased from 89% to 85%. GEN increased slightly from 92% to 93%, and IPM improved from 90% to 91%. MEM increased from 91% to 94%. NOR decreased from 90% to 86%. TZP remained stable at 98% for both years. Data for ceftolozane/tazobactam (CZT) were available only for 2019 and showed a high susceptibility of 99%.

**Figure 19:**
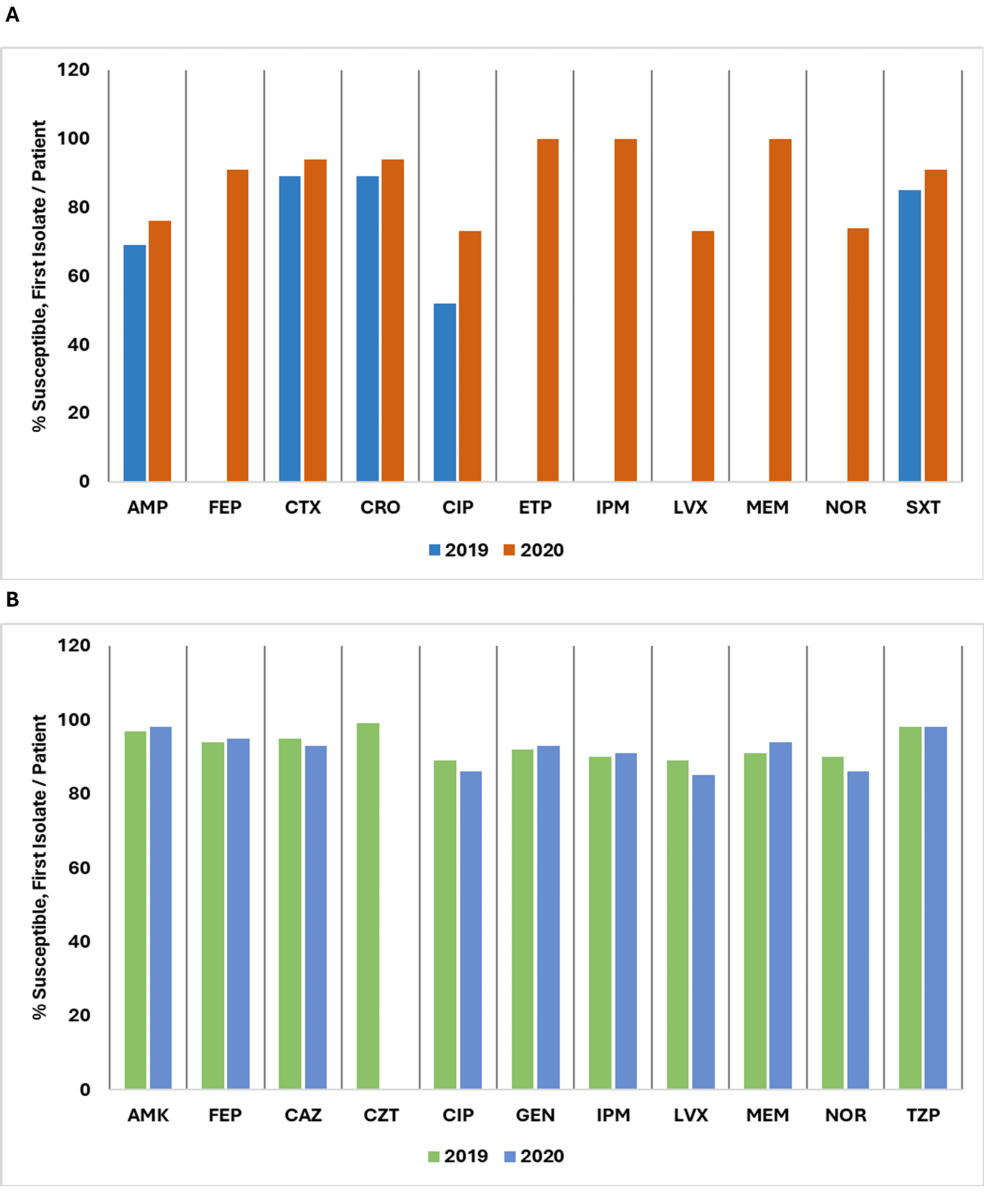
(A) Susceptibility (%) of Salmonella species: 2019 vs. 2020. (B) Susceptibility (%) of Pseudomonas species: 2019 vs. 2020. AMK: amikacin; AMP: ampicillin; CAZ: ceftazidime; CIP: ciprofloxacin; CRO: ceftriaxone; CTX: cefotaxime; CZT: ceftolozane/tazobactam; ETP: ertapenem; FEP: cefepime; GEN: gentamicin; IPM: imipenem; LVX: levofloxacin; MEM: meropenem; NOR: norfloxacin; SXT: trimethoprim/sulfamethoxazole; TZP: piperacillin/tazobactam. Source: Data from institutional antibiogram. Image credits: Kanika Vats.

Figure 19 shows a comparison of the percentage of susceptible isolates per patient for *Salmonella species*and *Pseudomonas species* between 2019 and 2020.

For *Acinetobacter species*, susceptibility remained high for AMK, stable at 99% across both years. FEP showed a slight decrease from 97% in 2019 to 95% in 2020, while CAZ decreased from 92% to 88%. CIP improved marginally from 91% to 92%. GEN increased from 97% to 98%, and IPM improved from 98% to 99%. LVX also showed an improvement from 91% to 92%. MEM remained high, increasing from 98% to 99%. Both TZP and SXT showed slight increases, with TZP going from 94% to 95% and SXT from 94% to 95%.

For *Haemophilus influenzae*, AMC dropped from 70% in 2019 to 56% in 2020. AMP was 71% in 2019. FEP, CFM, and CTX maintained 100% susceptibility. CRO, CXM, and GEN were high in 2019, with CIP improving to 100% in 2020. IPM increased to 100% in 2020. MEM remained at 100%. TZP was 99% in 2019, and TCY was 96%. SXT decreased to 78% in 2019.

**Figure 20:**
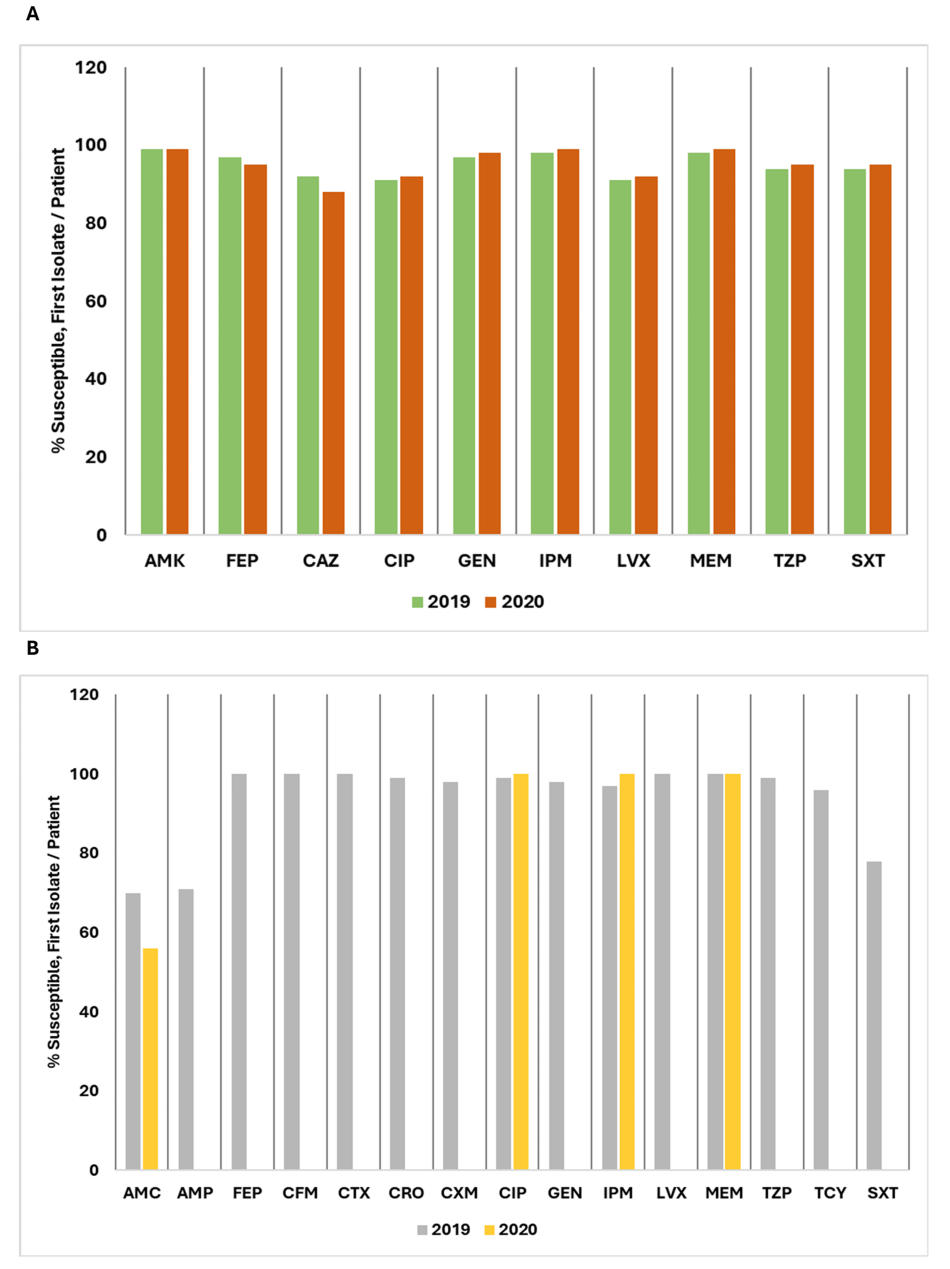
(A) Susceptibility (%) of Acinetobacter species: 2019 vs. 2020. (B) Susceptibility (%) of Haemophilus influenzae: 2019 vs. 2020. AMK: amikacin; AMC: amoxicillin/clavulanic acid; AMP: ampicillin; CXM: cefuroxime; CFM: cefixime; CAZ: ceftazidime; CIP: ciprofloxacin; CRO: ceftriaxone; CTX: cefotaxime; FEP: cefepime; GEN: gentamicin; IPM: imipenem; LVX: levofloxacin; MEM: meropenem; SXT: trimethoprim/sulfamethoxazole; TZP: piperacillin/tazobactam; TCY: tetracycline. Source: Data from institutional antibiogram. Image credits: Kanika Vats.

Figure 20 illustrates a comparison of the percentage of susceptible isolates per patient for *Acinetobacter species* and *Haemophilus influenzae* between 2019 and 2020.

Hence, these results showed that the COVID-19 pandemic notably impacted healthcare system performance, leading to increased hospital stays, a rise in HAIs, and higher readmission rates with associated financial costs. The pandemic also influenced antibiotic stewardship, with mixed outcomes. At the same time, some improvements were observed, and challenges such as extended hospital stays, increased use of probiotics, antidiarrheal treatments, and evolving resistance patterns in both Gram-positive and Gram-negative bacteria persisted. These trends underscore the need for enhanced stewardship practices, improved discharge planning, and strengthened infection control measures to mitigate AMR's ongoing impact on the healthcare system.

## Discussion

The Emirate of Abu Dhabi's health regulator has introduced guidelines for the ASP to improve antimicrobial prescribing practices and reduce the emergence and spread of resistant bacterial strains in healthcare settings and communities [16]. In response to the growing demand for rigorous reporting, the regulator has also implemented a new standard for monitoring and reporting AMR. This standard outlines the requirements for a laboratory-based AMR surveillance system in Abu Dhabi and specifies the responsibilities of healthcare facilities to gather, monitor, and report antimicrobial susceptibility testing (AST) results and related data [17]. Our results highlight the critical need for a robust monitoring and reporting framework to effectively address AMR.

A recent report on AMR trends in Abu Dhabi from 2010 to 2022 highlights a high and/or increasing level of antimicrobial resistance, particularly for *Staphylococcus aureus* (methicillin-resistant *Staphylococcus aureus*), extended-spectrum beta-lactamase (ESBL) *E. coli*, and ESBL *K. pneumoniae*, with rates notably exceeding those observed in western European countries. The report also identified critical priority pathogens for the region, including *Acinetobacter baumannii*, *Pseudomonas aeruginosa*, all *Enterobacterales*, *E. coli*, and *Klebsiella pneumoniae* [18]. These findings are consistent with our study results, underscoring the urgent need for comprehensive strategies to combat AMR effectively.

In July 2024, a report highlighted a 20% increase in antimicrobial resistance during the COVID-19 pandemic compared to pre-pandemic levels, peaking in 2021 and remaining elevated through 2022. Additionally, clinical cases of *Candida auris *- a resistant yeast associated with severe illness and spread in healthcare settings - rose nearly five-fold from 2019 to 2022. These findings emphasize the urgent need for strengthened efforts to address and mitigate antimicrobial resistance [19].

Extensive overuse of antibiotics during the COVID-19 pandemic has likely contributed to the "silent" spread of AMR. Despite only 8% of hospitalized COVID-19 patients having bacterial co-infections that required antibiotics, around 75% received antibiotics as a precaution. Antibiotic usage varied significantly, from 33% in the Western Pacific Region to 83% in the Eastern Mediterranean and African regions. Between 2020 and 2022, antibiotic prescriptions decreased in Europe and the Americas but increased in Africa. The highest rates of antibiotic use were seen among severe or critical COVID-19 cases, with a global average of 81%, while usage in mild or moderate cases varied, peaking at 79% in the African region [20]. In our study, we observed a similar trend of increased antibiotic use during the COVID-19 pandemic, aligning with global findings.

The study revealed an increase in the use of duplicate anaerobic antibiotics, contrary to guidelines that advise reviewing antibiotic therapy to avoid unnecessary duplication, particularly with agents that have overlapping spectra. Using two agents with anaerobic activity is generally redundant [21,22]. As a result, these prescribing practices should be reassessed.

Optimizing antibiotic use is vital for treating infections effectively, protecting patients from the negative consequences of unnecessary antibiotic use, and tackling antibiotic resistance. ASPs assist clinicians in improving patient outcomes and minimizing harm by refining their antibiotic prescribing practices [23,24]. These hospital-based programs are shown to boost infection cure rates, cut down on treatment failures and *Clostridium difficile* infections, reduce adverse effects, combat antibiotic resistance, and lower both hospital costs and lengths of stay [24-26].

Upon reviewing empirical therapy decisions through a comparison of microbiologic culture reports with MAR, several discrepancies were identified. These included continuing antibiotic treatment despite negative culture results, not following the organizational antibiogram for resistant antibiotics, and administering antibiotics without available susceptibility data from culture reports. The analysis revealed a notable increase in inaccurate empirical decisions, surpassing the roughly 30% of antibiotics deemed unnecessary or suboptimal in US acute care hospitals [23,27].

Therefore, a comprehensive understanding of proper antibiotic use is essential for preventing AMR. Despite the focus on COVID-19 and other emerging infectious diseases, AMR continues to be a significant, ongoing challenge. Like pandemics, AMR is a global issue with far-reaching consequences. Although its immediate impact on daily life may seem less pronounced, the potential long-term effects on humanity are profound. Consequently, it is crucial to investigate and implement strategies to manage and mitigate the rise of AMR at both local and global levels [28].

Our study identifies significant shifts in antibiotic use and resistance trends due to COVID-19, including increased use of duplicate anaerobic antibiotics despite guidelines. This underscores the need for better review processes and highlights the ongoing challenge of combating AMR, emphasizing the crucial role of effective ASPs.

Limitations

The retrospective nature of the study limits the ability to infer causality and may introduce biases inherent in such analyses. Dependence on electronic medical records could result in missing data, coding errors, and inconsistencies in record-keeping, potentially affecting data accuracy. The findings may not be generalizable beyond the specific healthcare settings and patient demographics studied. The focus on hospitalized patients and specific infections excludes outpatient data and a wider range of infection types. Furthermore, the inability to generate reports on staff involvement in patient care restricted the analysis of financial impacts on claims submitted to health insurance.

Future research should address these limitations by incorporating prospective study designs, expanding data sources to include outpatient settings, and exploring a broader range of infection types. Additionally, enhancing data collection methods and incorporating comprehensive reports on staff involvement and financial impacts could provide a more complete picture of the effectiveness and economic implications of ASPs.

The findings underscore the urgent need to enhance ASPs to address these trends effectively. The increase in inaccurate empirical therapy decisions, compared to established benchmarks in other regions, further emphasizes the need for reassessment and improvement of prescribing practices.

## Conclusions

The COVID-19 pandemic led to notable changes in antibiotic prescribing practices and resistance trends. Data revealed increased average hospital stay lengths, a rise in duplicate antibiotic use, and a shift toward empirical treatments despite insufficient supporting evidence. While there was an improvement in stewardship practices in 2020, significant challenges related to AMR persisted, including rising healthcare costs, increased mortality rates, inadequate surveillance, the spread of resistance, and the overuse and misuse of antibiotics. These findings underscore the need for ongoing efforts to strengthen antibiotic stewardship programs and enhance data-driven prescribing practices. Further research is crucial to better understand the broader impact of the pandemic on AMR and to develop strategies to optimize antibiotic use and improve patient outcomes.
